# Association of Maternal Autoimmune Diseases With Risk of Mental Disorders in Offspring in Denmark

**DOI:** 10.1001/jamanetworkopen.2022.7503

**Published:** 2022-04-15

**Authors:** Hua He, Yongfu Yu, Zeyan Liew, Mika Gissler, Krisztina D. László, Unnur Anna Valdimarsdóttir, Jun Zhang, Fei Li, Jiong Li

**Affiliations:** 1 Developmental and Behavioral Pediatric Department and Child Primary Care Department, Xinhua Hospital Affiliated to Shanghai Jiao Tong University School of Medicine, Shanghai, China; 2 Ministry of Education, Shanghai Key Laboratory of Children’s Environmental Health, Xinhua Hospital Affiliated to Shanghai Jiao Tong University School of Medicine, Shanghai, China; 3 Department of Biostatistics, School of Public Health and the Key Laboratory of Public Health Safety of Ministry of Education, Fudan University, Shanghai, China; 4 Department of Clinical Medicine, Department of Clinical Epidemiology, Aarhus University, Denmark; 5 Yale Center for Perinatal, Pediatric, and Environmental Epidemiology, Yale School of Public Health, New Haven, Connecticut; 6 Department of Environmental Health Sciences, Yale School of Public Health, New Haven, Connecticut; 7 Finnish Institute for Health and Welfare, Information Services Department, Helsinki, Finland; 8 Research Centre for Child Psychiatry, University of Turku, Turku, Finland; 9 Region Stockholm, Academic Primary Health Care Centre, Stockholm, Sweden; 10 Department of Molecular Medicine and Surgery, Karolinska Institute, Stockholm, Sweden; 11 Department of Global Public Health, Karolinska Institute, Stockholm, Sweden; 12 Center of Public Health Sciences, University of Iceland, Reykjavik, Iceland; 13 Department of Medical Epidemiology and Biostatistics, Karolinska Institute, Stockholm, Sweden

## Abstract

**Importance:**

Maternal immune activation during pregnancy is associated with increased risks of several mental disorders in offspring during childhood, but little is known about how maternal autoimmune diseases during pregnancy are associated with mental health in offspring during and after childhood.

**Objective:**

To investigate the association between maternal autoimmune diseases before childbirth and risk of mental disorders among offspring up to early adulthood.

**Design, Setting, and Participants:**

This population-based nationwide cohort study used data from Danish national registers on singletons born in Denmark from 1978 to 2015 with up to 38 years of follow-up. Data analyses were conducted from March 1, 2020, through September 30, 2021.

**Exposures:**

Maternal autoimmune disease diagnosed before or during pregnancy according to the Danish National Patient Register.

**Main Outcomes and Measures:**

The main outcome was mental disorders, defined by hospital diagnoses, in offspring. Cox proportional hazards regression was used to estimate hazard ratios (HRs) and 95% CIs for mental disorders.

**Results:**

Of the 2 254 234 singleton infants included in the study (median age, 16.7 years [IQR, 10.5-21.7 years]; 51.28% male), 2.26% were born to mothers with autoimmune diseases before childbirth. Exposed participants had an increased risk of overall mental disorders compared with their unexposed counterparts (HR, 1.16; 95% CI, 1.13-1.19; incidence, 9.38 vs 7.91 per 1000 person-years). Increased risks of overall mental disorders in offspring were seen in different age groups for type 1 diabetes (1-5 years: HR, 1.35 [95% CI, 1.17-1.57]; 6-18 years: HR, 1.24 [95% CI, 1.15-1.33]; >18 years: HR, 1.19 [95% CI, 1.09-1.30]) and rheumatoid arthritis (1-5 years: HR, 1.42 [95% CI, 1.16-1.74]; 6-18 years: HR, 1.19 [95% CI, 1.05-1.36]; >18 years: HR, 1.28 [95% CI, 1.02-1.60]). Regarding specific mental disorders, increased risk after exposure to any maternal autoimmune disorder was observed for organic disorders (HR, 1.54; 95% CI, 1.21-1.94), schizophrenia (HR, 1.35; 95% CI, 1.21-1.51), obsessive-compulsive disorder (HR, 1.42; 95% CI, 1.24-1.63), mood disorders (HR, 1.12; 95% CI, 1.04-1.21), and a series of neurodevelopmental disorders (eg, childhood autism [HR, 1.21; 95% CI, 1.08-1.36] and attention-deficit/hyperactivity disorder [HR, 1.19; 95% CI, 1.12-1.26]).

**Conclusions and Relevance:**

In this cohort study in Denmark, prenatal exposure to maternal autoimmune diseases was associated with increased risks of overall and type-specific mental disorders in offspring. Maternal type 1 diabetes and rheumatoid arthritis during pregnancy were associated with offspring’s mental health up to early adulthood. Individuals prenatally exposed to autoimmune disease may benefit from long-term surveillance for mental disorders.

## Key Points

**Question:**

Is prenatal maternal autoimmune disease associated with an increased long-term risk of mental disorders in offspring?

**Findings:**

In this cohort study of 2 254 234 singleton infants with up to 38 years of follow-up, prenatal maternal autoimmune diseases were associated with increased risks of a wide range of mental disorders in offspring. Maternal type 1 diabetes and rheumatoid arthritis during pregnancy were associated with offspring’s mental health up to early adulthood.

**Meaning:**

The findings suggest that offspring of mothers with autoimmune diseases may benefit from long-term surveillance for mental disorders.

## Teaser

This cohort study uses Danish national registry data to assess the association of maternal autoimmune diseases before childbirth with risk of mental disorders among offspring up to early adulthood.

## Introduction

The prevalence of autoimmune diseases is higher than 5%, with these diseases having a high burden.^1^ Most autoimmune diseases are more common in females predominantly during the childbearing years.^1,2^ Animal studies suggest that maternal immune activation can induce alterations in brain morphologic features, abnormal neurotransmitter activity, and behavioral deficits in offspring^3,4,5^; these alterations support a role of intrauterine exposure to immunologic insults in the cause of mental disorders. Empirical evidence has suggested an association between certain maternal autoimmune diseases (eg, type 1 diabetes, rheumatoid arthritis) and specific mental disorders (eg, autism spectrum disorder, attention-deficit/hyperactivity disorder [ADHD]) in offspring.^6,7,8,9,10,11,12,13,14,15^ However, most previous population-based studies were restricted to certain maternal autoimmune diseases and mental disorders in offspring during childhood and had small sample sizes. Using a Danish registry-based cohort of more than 2 million live-born singletons with up to 38 years of follow-up, we investigated the associations between a wide range of maternal autoimmune diseases diagnosed before childbirth and the risks of the full spectrum of mental disorders in offspring during childhood and early adulthood.

## Methods

This cohort study was approved by the Danish Data Protection Agency. By Danish law, no informed consent was required because this was a register-based study using anonymized data. The study followed the Strengthening the Reporting of Observational Studies in Epidemiology (STROBE) reporting guideline. Data analyses were performed from March 1, 2020, through September 30, 2021.

### Study Population

We conducted a population-based cohort study using data from Danish national registers.^16^ A total of 2 272 473 live-born singletons delivered in Denmark from 1978 to 2015 were identified from the Danish Medical Birth Registry.^17^ We excluded 18 239 infants who died or emigrated from Denmark before 1 year of age. The final cohort consisted of 2 254 234 births with 38 916 359 person-years.

### Assessment of Autoimmune Diseases

Offspring born to mothers diagnosed with autoimmune disease before childbirth were considered to have been prenatally exposed to maternal autoimmune disease. Information on autoimmune diseases was obtained from the Danish National Patient Register (NPR).^18^ The diagnostic systems used were the *International Classification of Diseases, Eighth Revision* (*ICD-8*) from 1969 to 1993 and the *International Statistical Classification of Diseases and Related Health Problems, Tenth Revision* (*ICD-10*) from 1994 onward. Using data from the NPR, information on 29 different autoimmune diseases in mothers of cohort members was retrieved (eTable 1 in the Supplement). The time of onset was defined as the first day of the first hospital contact for each autoimmune disease diagnosis. If a mother had a history of more than 1 autoimmune disease, the first date of the earliest diagnosed autoimmune disease was set as the onset time for any autoimmune disease. The first recorded date of diagnosis of autoimmune disease was used to assess whether the diagnosis was made before delivery.

### Mental Disorders in Offspring

Information on mental disorders was obtained from the Psychiatric Central Research Register and the NPR.^18,19^ The specific diagnoses included in each group of disorders according to *ICD-10* and *ICD-8* codes are presented in eTable 2 in the Supplement.^20,21^ For each specific mental disorder, the date of onset was defined as the first day of the first hospital contact. Individuals with more than 1 disorder were included in the numerator for each specific disorder.

### Potential Confounders

We included the following potential confounders: sex of the child (male or female), calendar period of birth (1978-1985, 1986-1994, 1995-2005, or 2006-2015), parental psychiatric history before childbirth (yes or no), and maternal characteristics, including parity (1, 2, or ≥3 births), maternal age at birth (<20, 20-24, 25-29, 30-34, or ≥35 years), highest attained educational level (≤9, 10-14, or ≥15 years), cohabitation with a partner (yes or no), residence (Copenhagen, city with ≥100 000 inhabitants, or other), and country of birth (Denmark, other). Missing data for each variable were coded separately.

### Statistical Analysis

Follow-up for offspring started at the earliest possible age at onset of the disorder (for each disorder separately) (eTable 2 in the Supplement) until the date of the first diagnosis, death, emigration, or December 31, 2016, whichever came first. Cox proportional hazards regression was used to estimate hazard ratios (HRs) with 95% CIs to assess the associations of interest, with offspring’s age as the time scale. Offspring born to mothers without any autoimmune disease diagnosis before delivery were treated as the reference group. Based on the main hypothesis in this study, the association between overall maternal autoimmune diseases and any mental disorder in offspring was the primary outcome analyzed. The log-minus-log survival curves were parallel (eFigure in the Supplement); thus, it was assumed that the proportional hazards assumption was not violated. We also estimated whether the overall risk of mental disorders in offspring associated with specific maternal autoimmune diseases differed by offspring’s age (≤5, 6-18, and >18 years).

In sensitivity analyses, we evaluated the associations by comorbidity of different types of autoimmune diseases. We excluded preterm births to assess whether the risk pattern was modified by fetal maturity in utero. In addition, we restricted the analyses to individuals without congenital malformations of the nervous system and chromosomal abnormalities to consider their potential associations with mental disorders. Because of the change in *ICD* version in 1994 and the extension of the NPR with data from 3 departments (inpatient, outpatient, and emergency) in 1995, we performed subanalyses restricted to individuals born after 1997. To explore potential residential confounding from genetic factors and shared familial factors, we used paternal autoimmune diseases as the negative control exposure and evaluated associations between paternal autoimmune diseases diagnosed before childbirth and mental disorders in children. To elucidate the role of maternal medication use for autoimmune diseases or mental disorders before or during pregnancy, we obtained information on medications by Anatomical Therapeutic Chemical codes from the Danish National Prescription Registry (eTable 3 in the Supplement).^22^ We adjusted for these variables in the Cox proportional hazards regression model. Because the Danish National Prescription Registry was established in 1995, only participants born between 1998 and 2015 were included. Also, we applied multiple imputation to address missing values and propensity score methods to validate the robustness of the results. All analyses were conducted using SAS, version 9.4 (SAS Institute).

## Results

Of the 2 254 234 singleton infants included (median age, 16.7 years [IQR, 10.5-21.7 years]; 1 155 982 [51.28%] male), 50 863 (2.26%) were born to mothers with autoimmune diseases diagnosed before or during pregnancy and 2 203 371 were born to mothers without autoimmune diseases (97.74%). Compared with mothers without autoimmune disease, mothers with autoimmune diseases were more likely to be older at the time of pregnancy, to have more than 1 child, to have higher educational level, and to live alone. Compared with unexposed offspring, offspring exposed to maternal autoimmune diseases were more likely to be born preterm (Table).

**Table.** Baseline Characteristics of the Offspring According to Prenatal Exposure to Maternal Autoimmune Diseases

Characteristics	Maternal autoimmune disease, No. (%)	
	Exposed offspring (n = 50 863)	Unexposed offspring (n = 2 203 371)
Sex		
Female	24 997 (49.15)	1 072 255 (48.66)
Male	25 863 (50.85)	1 130 119 (51.29)
Missing	3 (0.01)	997 (0.05)
Calendar year of birth		
1978-1985	3187 (6.27)	424 492 (19.27)
1986-1994	6232 (12.25)	538 798 (24.45)
1995-2005	16 391 (32.23)	679 775 (30.85)
2006-2016	25 053 (49.26)	560 306 (25.43)
Gestational age at birth, wk		
<37	5161 (10.15)	94 899 (4.31)
≥37	44 991 (88.46)	2 028 343 (92.06)
Missing	711 (1.40)	80 129 (3.64)
Maternal parity		
1	21 193 (41.67)	986 635 (44.78)
2	19 583 (38.50)	818 434 (37.14)
≥3	10 087 (19.83)	398 302 (18.08)
Maternal age at childbirth, y		
<20	332 (0.65)	39 822 (1.81)
20-34	38 590 (75.87)	1 839 428 (83.48)
≥35	11 941 (23.48)	324 121 (14.71)
Maternal educational level at childbirth, y		
0-9	10 902 (21.43)	595 282 (27.02)
10-14	22 309 (43.86)	951 005 (43.16)
≥15	17 136 (33.69)	613 715 (27.85)
Missing	516 (1.01)	43 369 (1.97)
Maternal cohabitation at childbirth		
Yes	27 003 (53.09)	1 214 548 (55.12)
No	23 851 (46.89)	986 215 (44.76)
Missing	9 (0.02)	2608 (0.12)
Maternal residence at childbirth		
Copenhagen	6219 (12.23)	244 865 (11.11)
City with ≥100 000 inhabitants	7110 (13.98)	280 727 (12.74)
Other	37 534 (73.79)	1 677 779 (76.15)
Maternal country of birth		
Denmark	46 124 (90.68)	1 955 104 (88.73)
Other	4712 (9.26)	243 512 (11.05)
Missing	27 (0.05)	4755 (0.22)

**Figure 1.**
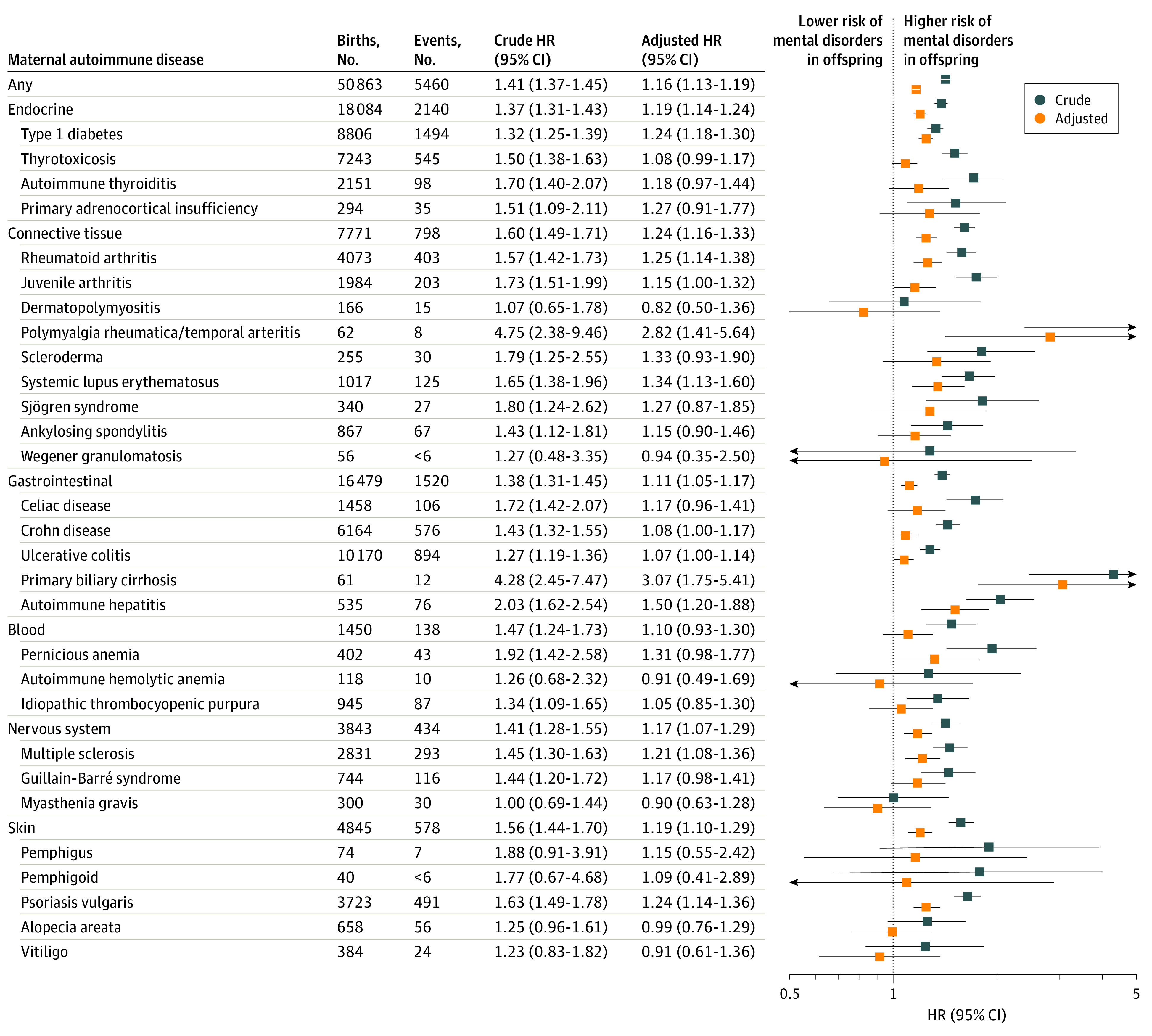
Associations Between Specific Maternal Autoimmune Diseases Diagnosed Before Childbirth and Risk of Overall Mental Disorders in Offspring In the adjusted model, the associations were controlled for parental psychiatric history, maternal characteristics (parity, age at birth, highest educational level, cohabitation with a partner, residence, and birth country), and birth characteristics (child’s sex, calendar year of birth). Markers represent estimates, with horizontal lines indicating 95% CIs. HR indicates hazard ratio.

**Figure 2.**
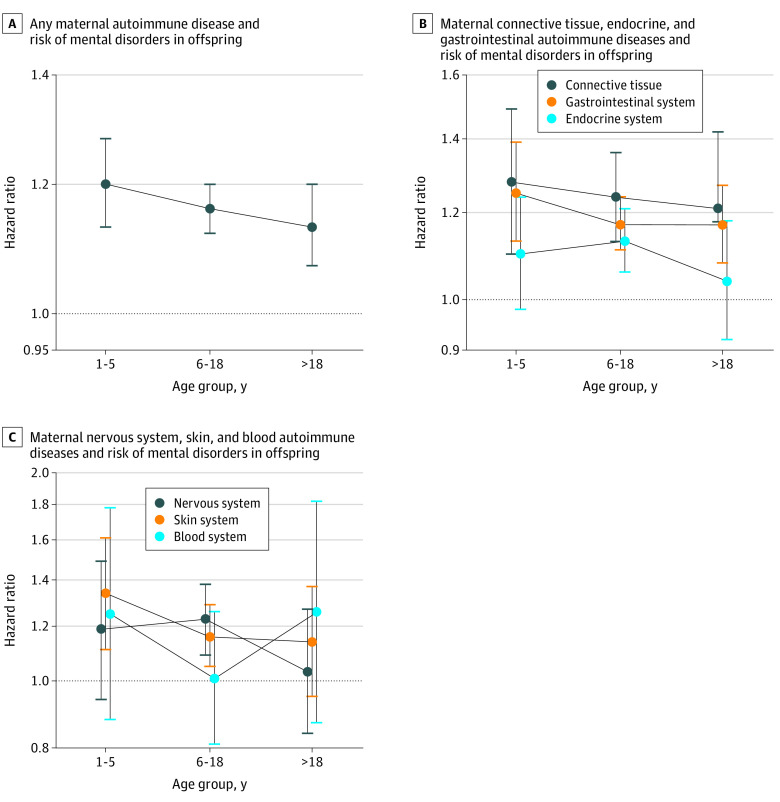
Associations Between Offspring’s Overall Mental Disorders and Maternal Organ System–Specific Autoimmune Diseases by Offspring’s Age Hazard ratios were adjusted for parental psychiatric history, maternal characteristics (parity, age at birth, highest educational level, cohabitation with a partner, residence, and birth country), and birth characteristics (child’s sex, calendar year of birth). Error bars indicate 95% CIs.

**Figure 3.**
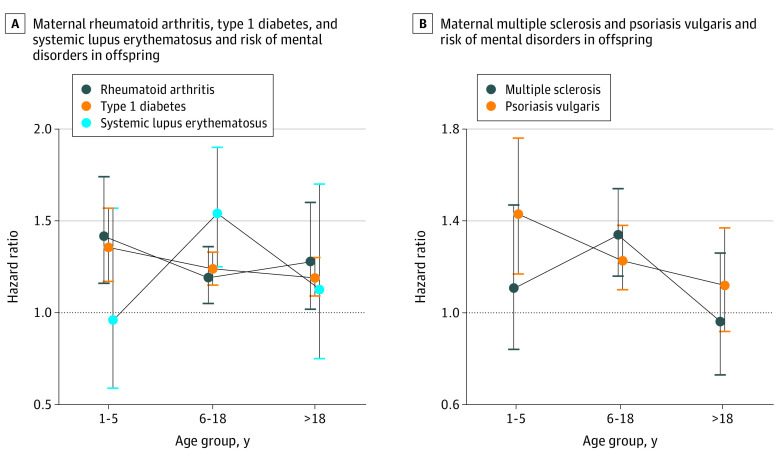
Associations Between Offspring’s Overall Mental Disorders With 5 Common Maternal Autoimmune Diseases by Offspring Age Hazard ratios were adjusted for parental psychiatric history, maternal characteristics (parity, age at birth, highest educational level, cohabitation with a partner, residence, and birth country), and birth characteristics (child’s sex, calendar year of birth). Error bars indicate 95% CIs.

During the follow-up period of up to 38 years (median, 17 years [IQR, 8-25 years]), 5460 offspring of mothers with autoimmune diseases (10.73%) and 303 092 offspring of mothers without autoimmune diseases (13.76%) were diagnosed with a mental disorder; the incidence of any mental disorder was higher among exposed offspring (9.38 per 1000 person-years) compared with unexposed offspring (7.91 per 1000 person-years) (Figure 1). Overall, offspring exposed to any maternal autoimmune disease had a 16% higher risk of overall mental disorders than did unexposed offspring (HR, 1.16; 95% CI, 1.13-1.19). When we classified autoimmune disease according to organ system, increased risk of developing any mental disorder in children was observed for most system types of maternal autoimmune diseases (connective tissue system: HR, 1.24 [95% CI, 1.16-1.33]; endocrine system: HR, 1.19 [1.14-1.24]; gastrointestinal system: HR, 1.11 [95% CI, 1.05-1.17]; blood system: HR, 1.10 [95% CI, 0.93-1.30]; nervous system: HR, 1.17 [95% CI, 1.07-1.29]; skin system: HR, 1.19 [95% CI, 1.10-1.29]). Specifically, the highest overall risk for mental disorders in offspring was observed for primary biliary cirrhosis (HR, 3.07; 95% CI, 1.75-5.41) but with small numbers of events (12 of 61 births). The 5 most common maternal autoimmune diseases associated with the overall risk of mental disorders in offspring were type 1 diabetes (HR, 1.24; 95% CI, 1.18-1.30), rheumatoid arthritis (HR, 1.25; 95% CI, 1.14-1.38), systemic lupus erythematosus (HR, 1.34; 95% CI, 1.13-1.60), multiple sclerosis (HR, 1.21; 95% CI, 1.08-1.36), and psoriasis vulgaris (HR, 1.24; 95% CI,1.14-1.36). Those associations existed in each age group for any autoimmune disease (1-5 years: HR, 1.20 [95% CI, 1.13-1.28]; 6-18 years: HR, 1.16 [95% CI, 1.12-1.20]; >18 years: HR, 1.13 [95% CI, 1.07-1.20]), endocrine system (1-5 years: HR, 1.25 [95% CI, 1.13-1.39]; 6-18 years: HR, 1.17 [95% CI,1.11-1.24]; >18 years: HR, 1.17 [95% CI, 1.08-1.27]), and connective tissue autoimmune diseases (1-5 years: HR, 1.28 [95% CI, 1.10-1.49]; 6-18 years: HR, 1.24 [95% CI, 1.13-1.36]; 18 years: HR, 1.21 [95% CI, 1.02-1.42]), type 1 diabetes (1-5 years: HR, 1.35 [95% CI, 1.17-1.57]; 6-18 years: HR, 1.24 [95% CI, 1.15-1.33]; >18 years: HR, 1.19 [95% CI, 1.09-1.30]), and rheumatoid arthritis (1-5 years: HR, 1.42 [95% CI, 1.16-1.74]; 6-18 years: HR, 1.19 [95% CI, 1.05-1.36]; >18 years: HR, 1.28 [95% CI, 1.02-1.60]) but differed by age groups for systemic lupus erythematosus, multiple sclerosis, and psoriasis vulgaris (Figure 2 and Figure 3 and eTable 4 in the Supplement).

**Figure 4.**
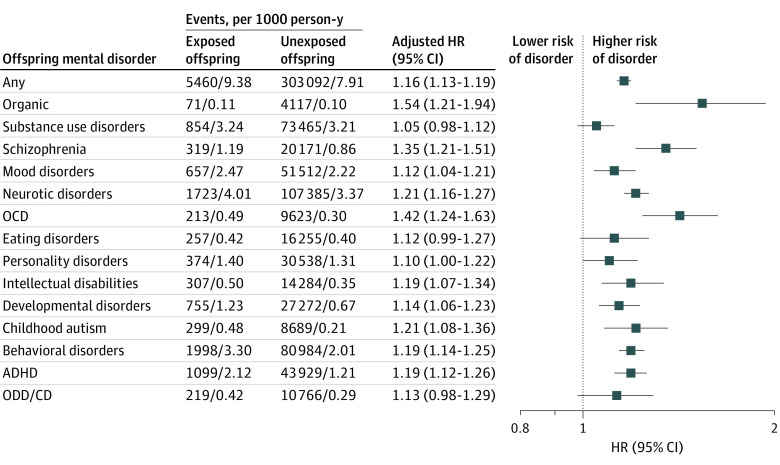
Association of Offspring’s Risk of Specific Mental Disorders With Prenatal Exposure to Any Autoimmune Disease Hazard ratios were adjusted for parental psychiatric history, maternal characteristics (parity, age at birth, highest educational level, cohabitation with a partner, residence, and birth country), and birth characteristics (adult’s sex, calendar year of birth). Markers represent estimates, with horizontal lines indicating 95% CIs. ADHD indicates attention-deficit/hyperactivity disorder; OCD, obsessive-compulsive disorder; ODD/CD, oppositional defiant disorder/conduct disorder.

Figure 4 shows the HRs for all studied categories of mental disorders in offspring after prenatal exposure to any maternal autoimmune disease compared with their unexposed counterparts. Higher risk was observed for organic disorders (HR, 1.54; 95% CI, 1.21-1.94), obsessive-compulsive disorder (HR, 1.42; 95% CI, 1.24-1.63), schizophrenia (HR, 1.35; 95% CI, 1.21-1.51), and mood disorders (HR, 1.12; 95% CI, 1.04-1.21). Offspring with maternal autoimmune disease history had slightly increased risks for a wide range of neurodevelopmental disorders, for example, intellectual disability (HR, 1.19; 95% CI, 1.07-1.34), childhood autism (HR, 1.21; 95% CI, 1.08-1.36), and ADHD (HR, 1.19; 95% CI, 1.12-1.26). The number of comorbid maternal autoimmune diseases did not substantially modify the increased risk for overall or specific mental disorders in offspring (eTable 5 in the Supplement).

In sensitivity analysis, effect estimates from analyses excluding preterm births were mostly consistent with those observed in the main analyses (eTables 6 and 7 in the Supplement). Results from separate analyses restricted to individuals without diagnoses of congenital malformations of the nervous system or chromosomal abnormalities or those who were born after 1997 were similar to those obtained in the primary analyses (eTables 8-11 in the Supplement). In addition, except for type 1 diabetes and psoriasis, most paternal autoimmune diseases diagnosed before the child was born were not significantly associated with the risk of overall mental disorders in offspring (eTable 12 in the Supplement). Increased risks were observed for organic disorders, schizophrenia, and obsessive-compulsive disorder in children with fathers with diagnoses of autoimmune diseases before the child was born (eTable 13 in the Supplement) but not for mood disorders or a wide range of neurodevelopmental disorders (eg, intellectual disability, childhood autism, and ADHD) compared with mothers who had autoimmune disorders (Figure 4). The results remained similar to those obtained in the primary analyses after adjusting for medication used before or during pregnancy (eTables 14 and 15 in the Supplement). The results from multiple imputation with 10 replications and propensity score methods yielded results similar to those of primary analyses (eTables 16-19 in the Supplement). In addition, we observed that children born to mothers with autoimmune diseases diagnosed before childbirth had an 11% increased risk of suicide, but the result was not statistically significant (HR, 1.11; 95% CI, 0.79-1.57) (eTable 20 in the Supplement).

## Discussion

This nationwide population-based study involving births of 2 254 234 singleton infants provided estimates regarding the associations between prenatal exposure to various maternal autoimmune diseases and the full spectrum of mental disorders in offspring. Maternal autoimmune disease diagnosed before childbirth was associated with a 16% increased overall risk of mental disorders in offspring. Prenatal exposure to most common specific autoimmune diseases, such as type 1 diabetes, rheumatoid arthritis, systemic lupus erythematosus, multiple sclerosis, and psoriasis, was associated with a significantly increased overall risk of mental disorders in offspring; the association of type 1 diabetes and rheumatoid arthritis with this risk lasted into early adulthood. In particular, children of mothers with autoimmune diseases diagnosed before delivery were more likely to develop a wide range of neurodevelopmental disorders (eg, intellectual disability, childhood autism, and ADHD), organic disorders, obsessive-compulsive disorder, schizophrenia, and mood disorders.

To our knowledge, this is the first study to analyze the association between a wide range of maternal autoimmune diseases diagnosed before childbirth and offspring’s full spectrum of mental disorders. The observed increased overall risk in offspring was apparent for most specific autoimmune diseases but varied (eg, primary biliary cirrhosis: HR, 3.07 [95% CI, 1.75-5.41] multiple sclerosis: HR, 1.21 [95% CI, 1.08-1.36]) perhaps because of the differences in pathogenicity or the degree of autoimmunity among diverse autoimmune diseases^23^ and statistical variance owing to small numbers of events (eg, 12 of 61 births with maternal primary biliary cirrhosis). Our findings corroborate the results of previous studies, suggesting an association of maternal autoimmune diseases with offspring’s mental disorders.^7,9,10^ However, direct comparisons between findings from earlier studies and our study are difficult because of differences in study design, case definition, and prevalence of autoimmune diseases in various regions. Of note, most of the previous investigations did not consider the temporal association between maternal autoimmune diseases and offspring’s mental disorders (ie, that maternal autoimmune diseases should be diagnosed before childbirth). Although some longitudinal studies have taken this into account,^15,24,25^ failure to limit the period of exposure before childbirth, together with lack of consideration for adverse birth outcomes (eg, preterm birth), are the main limitations of those earlier studies. Our study adds important epidemiological evidence to support the proposed maternal immune activation mechanism found in previous animal research.^3,4,5^

Most previous studies have focused on maternal autoimmune diseases and risks of selected specific mental disorders, and the results have been inconsistent.^7,8,10,11,13,15,25,26,27,28^ To our knowledge, no previous study has considered simultaneously a wide range of maternal autoimmune diseases and a large spectrum of mental disorders in offspring. In this study, we evaluated risks of the full spectrum of mental disorders in offspring born to mothers with autoimmune diseases. We found that prenatal exposure to maternal autoimmune diseases was associated with modestly elevated risks of neurodevelopmental disorders in childhood (eg, intellectual disabilities: HR, 1.19 [95% CI, 1.07-1.34]; childhood autism: HR, 1.21 [95% CI, 1.08-1.36]; ADHD: HR, 1.19 [95% CI, 1.12-1.26]). Previous reports have noted that maternal autoimmune diseases were more common among children with autism than among healthy control individuals, suggesting a possible association between autism and maternal autoimmune disease history.^7,28^ However, a nested case-control study that collected data on any autoimmune disease diagnosis in mothers in the 2 years before and after pregnancy found no such association.^29^ A limited number of studies focused on ADHD, another common neurodevelopmental disorder in childhood.^9,14,30,31^ Our study extended the finding of a recent hybrid study in which ADHD risk in offspring was the main outcome of interest and both original data from a cohort and meta-analyses of published studies were included.^14^ Results from a Danish study suggested that mothers with an autoimmune disease were more likely to have a child with poorer school performance than their unexposed counterparts,^12^ suggesting a potential association of maternal autoimmune activity with fetal brain development. To our knowledge, no previous study investigated the direct association between maternal autoimmune diseases and offspring’s risk of intellectual disability. However, direct comparisons and interpretations of these findings are hampered by different definitions of the exposure time window for maternal autoimmune diseases and limitations in study design, including small sample sizes and reliance on self-reported history of autoimmune diseases. Our study provides further evidence that maternal history of autoimmune disease before childbirth may be associated with increased risks of many other mental outcomes, such as organic disorders, mood disorders, and obsessive-compulsive disorder.

Several potential mechanisms have been proposed. Autoimmune diseases and mental disorders are heritable disorders and may share common genetic risk factors.^32^ Another alternative hypothesis could be that mothers who had disturbed autoimmunity before giving birth may transfer autoantibodies to the fetus during pregnancy.^33^ These transferred antibodies may activate the immune system in the child and alter normal brain development.^34,35^ Our finding that children born to mothers with autoimmune diseases diagnosed before childbirth had a higher risk of mental disorders would support the maternal transmission hypothesis. Previous studies have suggested that women with autoimmune disease have an increased risk of developing certain obstetric complications (eg, preterm births),^36,37,38^ which in turn may be associated with increased risk of mental disorders in the child.^39,40,41^ However, in analyses restricted to term births, the risk of mental disorders remained increased, suggesting that the associations cannot be explained entirely by these factors. Further studies are needed to gain a better understanding of the underlying mechanisms.

### Strengths and Limitations

Our study has several strengths. First, the prospectively collected registry data including all live births in Denmark minimized the potential selection bias and recall bias. Second, a large sample size of more than 2 million births provided sufficient statistical power to perform detailed subgroup analyses that, to our knowledge, have not been done previously. Third, the availability of sociodemographic and clinical information enabled consideration of a wide range of important confounding factors.

This study also has limitations. First, we lacked information on the potential exacerbation of autoimmune disease activity during pregnancy. The effect of autoimmune disease activity during pregnancy on the fetal environment and the potential transfer of maternal antibodies to the fetus are relevant and should be investigated in further studies. Second, we cannot rule out detection bias in our study because mothers with chronic autoimmune disease may use health care more frequently and, consequently, may more readily seek care for their children. Third, the study period spanned almost 4 decades, and advances in medical care over time and changes in the diagnostic criteria may have influenced the exposures and outcomes. However, adjustment for calendar time in the analyses would partially alleviate the effects of temporal changes in medical care. Fourth, we were not able to investigate the risk of mental disorders in late adulthood because the maximum follow-up was 38 years; however, to our knowledge, this study had the longest follow-up period for investigating maternal autoimmune diseases and offspring mental disorders. Fifth, the absence of information from primary care and the delayed inclusion of outpatient records in the Psychiatric Central Research Register and the NPR might have resulted in underestimation of the associations. This concern was partially addressed in our sensitivity analyses restricted to individuals born after 1997, when outpatient records were also included in the NPR and the Psychiatric Central Register; that analysis revealed similar results.

## Conclusions

In this large Danish population-based cohort study, we found that in utero exposure to a maternal autoimmune disease was associated with increased risks of overall and most type-specific mental disorders in offspring. These risks varied according to different subtypes of autoimmune diseases and specific mental disorders. Maternal type 1 diabetes and rheumatoid arthritis before childbirth were associated with overall mental disorders in offspring up to early adulthood. These findings suggest that a wide spectrum of mental disorders should be monitored in offspring of mothers with autoimmune diseases before or during pregnancy and especially for certain maternal autoimmune diseases (eg, type 1 diabetes and rheumatoid arthritis). Individuals prenatally exposed to autoimmune disease may benefit from long-term surveillance for mental disorders. If confirmed in future studies, our findings may have important implications for early screening and appropriate intervention for children at high risk of mental disorders.

## Notes

Supplement. **eTable 1.** Diagnostic Classification of Autoimmune Diseases Considered According to the *ICD-10* and Equivalent *ICD-8* Diagnoses **eTable 2.** Diagnostic Classification of Mental Disorders Considered According to the *ICD-10* and Equivalent *ICD-8* Diagnoses **eTable 3.** Anatomical Therapeutic Chemical (ATC) Codes for Autoimmune Disease and Mental Disorders Medications **eTable 4.** Associations Between Specific Maternal Autoimmune Diseases and Risk of Any Mental Disorder in the Offspring by Offspring’s Age **eTable 5.** Hazard Ratios of Any Mental Disorder Among the Offspring Stratified by the Numbers of Comorbid Maternal Autoimmune Diseases **eTable 6.** Associations Between Specific Maternal Autoimmune Diseases Diagnosed Before Childbirth and Overall Mental Disorders Among Term-Born Individuals **eTable 7.** Hazard Ratios of Specific Mental Disorders According to Prenatal Exposure to Any Maternal Autoimmune Disease Among Term-Born Individuals **eTable 8.** Associations Between Specific Maternal Autoimmune Diseases Diagnosed Before Child Birth and Overall Mental Disorders Among Individuals Without Congenital Malformations of the Nervous System or Chromosomal Abnormalities **eTable 9.** Hazard Ratios of Specific Mental Disorder According to Prenatal Exposure to Any Maternal Autoimmune Disease Among Individuals Without Congenital Malformations of the Nervous System and Chromosomal Abnormalities **eTable 10.** Associations Between Specific Autoimmune Diseases of Mothers and Overall Mental Disorders in the Offspring Born During 1998-2007 **eTable 11.** Associations Between Specific Autoimmune Diseases of Mothers and Overall Mental Disorders in the Offspring Born During 2008-2015 **eTable 12.** Associations Between Specific Paternal Autoimmune Diseases Diagnosed Before Childbirth and Overall Mental Disorders in Offspring **eTable 13.** Hazard Ratios of Specific Mental Disorder in Offspring According to Prenatal Exposure to Any Paternal Autoimmune Disease Diagnosed Before Childbirth **eTable 14.** Associations Between Specific Maternal Autoimmune Diseases Diagnosed Before Childbirth and Overall Mental Disorders, Adjusted for Medications Used by Mothers Before Childbirth in Addition to the Variables in the Main Model **eTable 15.** Hazard Ratios of Specific Mental Disorder According to Prenatal Exposure to Any Maternal Autoimmune Disease, Adjusted for Medications Used by Mothers Before Childbirth in Addition to the Variables in the Main Model **eTable 16.** Associations Between Specific Maternal Autoimmune Diseases Diagnosed Before Childbirth and Overall Mental Disorders in Offspring, With Multiple Imputation for Missing Data **eTable 17.** Hazard Ratios of Specific Mental Disorder According to Prenatal Exposure to Any Maternal Autoimmune Disease, With Multiple Imputation for Missing Data **eTable 18.** Associations Between Specific Maternal Autoimmune Diseases Diagnosed Before Childbirth and Overall Mental Disorders in Offspring, Using Propensity Score Methods **eTable 19.** Hazard Ratios of Specific Mental Disorder Among Individuals Born to Mothers With Any Autoimmune Disease, Using Propensity Score Methods **eTable 20.** Association Between Overall Maternal Autoimmune Diseases Diagnosed Before Childbirth and Suicide in Offspring **eFigure.** The Log-Minus-Log Survival Curve
