# Supplementary material for: Association of Maternal Autoimmune Diseases With Risk of Mental Disorders in Offspring in Denmark
Source: JAMA Netw Open. 2022 Apr 15;5(4):e227503. doi: 10.1001/jamanetworkopen.2022.7503 (PMC9012963; doi:10.1001/jamanetworkopen.2022.7503)
Supplement: Supplement. — eTable 1. Diagnostic Classification of Autoimmune Diseases Considered According to the ICD-10 and Equivalent ICD-8 Diagnoses eTable 2. Diagnostic Classification of Mental Disorders Considered According to the ICD-10 and Equivalent ICD-8 Diagnoses eTable 3. Anatomical Therapeutic Chemical (ATC) Codes for Autoimmune Disease and Mental Disorders Medications eTable 4. Associations Between Specific Maternal Autoimmune Diseases and Risk of Any Mental Disorder in the Offspring by Offspring’s Age eTable 5. Hazard Ratios of Any Mental Disorder Among the Offspring Stratified by the Numbers of Comorbid Maternal Autoimmune Diseases eTable 6. Associations Between Specific Maternal Autoimmune Diseases Diagnosed Before Childbirth and Overall Mental Disorders Among Term-Born Individuals eTable 7. Hazard Ratios of Specific Mental Disorders According to Prenatal Exposure to Any Maternal Autoimmune Disease Among Term-Born Individuals eTable 8. Associations Between Specific Maternal Autoimmune Diseases Diagnosed Before Child Birth and Overall Mental Disorders Among Individuals Without Congenital Malformations of the Nervous System or Chromosomal Abnormalities eTable 9. Hazard Ratios of Specific Mental Disorder According to Prenatal Exposure to Any Maternal Autoimmune Disease Among Individuals Without Congenital Malformations of the Nervous System and Chromosomal Abnormalities eTable 10. Associations Between Specific Autoimmune Diseases of Mothers and Overall Mental Disorders in the Offspring Born During 1998-2007 eTable 11. Associations Between Specific Autoimmune Diseases of Mothers and Overall Mental Disorders in the Offspring Born During 2008-2015 eTable 12. Associations Between Specific Paternal Autoimmune Diseases Diagnosed Before Childbirth and Overall Mental Disorders in Offspring eTable 13. Hazard Ratios of Specific Mental Disorder in Offspring According to Prenatal Exposure to Any Paternal Autoimmune Disease Diagnosed Before Childbirth eTable 14. Associations Between Specific [file jamanetwopen-e227503-s001.pdf]

Supplementary Online Content

He H, Yu Y, Liew Z, et al. Association of maternal autoimmune diseases with risk of mental disorders in offspring in Denmark. *JAMA Netw Open*. 2022;5(4):e227503. doi:10.1001/jamanetworkopen.2022.7503

eTable 1. Diagnostic Classification of Autoimmune Diseases Considered According to the *ICD-10* and Equivalent *ICD-8* Diagnoses
eTable 2. Diagnostic Classification of Mental Disorders Considered According to the *ICD-10* and Equivalent *ICD-8* Diagnoses
eTable 3. Anatomical Therapeutic Chemical (ATC) Codes for Autoimmune Disease and Mental Disorders Medications
eTable 4. Associations Between Specific Maternal Autoimmune Diseases and Risk of Any Mental Disorder in the Offspring by Offspring's Age
eTable 5. Hazard Ratios of Any Mental Disorder Among the Offspring Stratified by the Numbers of Comorbid Maternal Autoimmune Diseases
eTable 6. Associations Between Specific Maternal Autoimmune Diseases Diagnosed Before Childbirth and Overall Mental Disorders Among Term-Born Individuals
eTable 7. Hazard Ratios of Specific Mental Disorders According to Prenatal Exposure to Any Maternal Autoimmune Disease Among Term-Born Individuals
eTable 8. Associations Between Specific Maternal Autoimmune Diseases Diagnosed Before Child Birth and Overall Mental Disorders Among Individuals Without Congenital Malformations of the Nervous System or Chromosomal Abnormalities
eTable 9. Hazard Ratios of Specific Mental Disorder According to Prenatal Exposure to Any Maternal Autoimmune Disease Among Individuals Without Congenital Malformations of the Nervous System and Chromosomal Abnormalities
eTable 10. Associations Between Specific Autoimmune Diseases of Mothers and Overall Mental Disorders in the Offspring Born During 1998-2007
eTable 11. Associations Between Specific Autoimmune Diseases of Mothers and Overall Mental Disorders in the Offspring Born During 2008-2015

© 2022 He H et al. *JAMA Network Open*.

eTable 12. Associations Between Specific Paternal Autoimmune Diseases Diagnosed Before Childbirth and Overall Mental Disorders in Offspring
eTable 13. Hazard Ratios of Specific Mental Disorder in Offspring According to Prenatal Exposure to Any Paternal Autoimmune Disease Diagnosed Before Childbirth
eTable 14. Associations Between Specific Maternal Autoimmune Diseases Diagnosed Before Childbirth and Overall Mental Disorders, Adjusted for Medications Used by Mothers Before Childbirth in Addition to the Variables in the Main Model
eTable 15. Hazard Ratios of Specific Mental Disorder According to Prenatal Exposure to Any Maternal Autoimmune Disease, Adjusted for Medications Used by Mothers Before Childbirth in Addition to the Variables in the Main Model
eTable 16. Associations Between Specific Maternal Autoimmune Diseases Diagnosed Before Childbirth and Overall Mental Disorders in Offspring, With Multiple Imputation for Missing Data
eTable 17. Hazard Ratios of Specific Mental Disorder According to Prenatal Exposure to Any Maternal Autoimmune Disease, With Multiple Imputation for Missing Data
eTable 18. Associations Between Specific Maternal Autoimmune Diseases Diagnosed Before Childbirth and Overall Mental Disorders in Offspring, Using Propensity Score Methods
eTable 19. Hazard Ratios of Specific Mental Disorder Among Individuals Born to Mothers With Any Autoimmune Disease, Using Propensity Score Methods
eTable 20. Association Between Overall Maternal Autoimmune Diseases Diagnosed Before Childbirth and Suicide in Offspring
eFigure. The Log-Minus-Log Survival Curve

This supplementary material has been provided by the authors to give readers additional information about their work.

.

© 2022 He H et al. *JAMA Network Open*.

eTable 1. **Diagnostic classification of autoimmune diseases considered according to the ICD-10 and equivalent ICD-8 diagnoses.**

**Diagnoses of ADs**	**ICD8**	**ICD10**
Endocrine autoimmune diseases		
Type 1 Diabetes	249	E10
Thyreotoxicosis	242.00	E05.0
Autoimmune thyroiditis	245.03	E06.3
Primary adrenocortical insufficiency	255.1	E27.1
Connective tissue autoimmune diseases		
Rheumatoid arthritis	712.19, 712.39, 712.59	M05–M06
Juvenile arthritis	712.09	M08
Dermatopolymyositis	716	M33
Polymyalgia rheumatica/temporal arteritis	446.30–31, 446.39	M31.5–6, M35.3
Scleroderma	734.0	M34
Systemic lupus erythematosus	734.19	M32.1, M32.9
Sjögren syndrome	734.90	M35.0
Ankylosing Spondylitis	712.49	M45
Wegener granulomatosis	446.29	M31.3
Gastrointestinal autoimmune diseases		
Celiac disease	269.00	K90.0
Crohn disease	563.01	K50
Ulcerative colitis	563.19	K51
Primary biliary cirrhosis	571.90	K74.3
Autoimmune hepatitis	571.93	K73
Blood autoimmune diseases		
Pernicious anemia	281.0	D51.0
Autoimmune hemolytic anemia	283.90–91	D59.1
Idiopathic thrombocyopenic purpura	446.49, 287.10	D69.3
Nervous system autoimmune diseases		
Multiple sclerosis	340	G35
Guillanian-Barré syndrome	354	G61.0
Myasthenia gravis	733.09	G70.0
Skin autoimmune diseases		
Pemphigus	694 (×694.05)	L10
Pemphigoid	694.05	L12
Psoriasis vulgaris	696.09–10, 696.19	L40 (×L40.4)
Alopecia areata	704.00	L63
Vitiligo	709.01	L80.9

ICD-10: International Classification of Diseases, 10th revision (1994-2016); ICD-8: Danish modification of the International Classification of Diseases, 8th revision (1978-1993)

© 2022 He H et al. *JAMA Network Open.*

eTable 2. **Diagnostic classification of mental disorders considered according to the ICD-10 and equivalent ICD-8 diagnoses.**

Diagnoses	Abbreviated name	ICD-8	ICD-10	Earliest possible age at onset (years)
**Any mental disorder**	Any mental disorder	290 – 315	F00 – F99	1
**Organic, including symptomatic, mental disorders** *Includes dementia in Alzheimer's disease, vascular dementia, etc.*	Organic disorders	290·09, 290·10, 290·11, 290·18, 290·19, 292·x9, 293·x9, 294·x9, 309·x9	F00 – F09	1
**Mental and behavioral disorders due to psychoactive substance use** *Includes use of alcohol, cannabis, cocaine, nicotine, opioids, sedatives, hypnotics, anxiolytics, etc.*	Substance use disorders	291·x9, 294·39, 303·x9, 303·20, 303·28, 303·90, 304·x9	F10 – F19	10
**Schizophrenia and related disorders** *Includes schizophrenia, schizotypal disorders, schizoaffective disorders and other psychotic disorders.*	Schizophrenia	295·x9, 296·89, 297·x9, 298·29-298·99, 299·04, 299·05, 299·09, 301·83	F20 – F29	10
**Mood disorders** *Includes bipolar disorder, depressive disorders, etc*	Mood disorders	296·x9 (excluding 296·89), 298·09, 298·19, 300·49, 301·19	F30 – F39	10
**Neurotic, stress-related, and somatoform disorders** *Includes anxiety disorders, phobias, obsessive compulsive disorders, etc.*	Neurotic disorders	300·x9 (excluding 300·49), 305·x9, 305·68, 307·99	F40 – F48	5
**Obsessive-compulsive disorder**	OCD	300.39	F42	5
**Eating disorders** *Includes anorexia nervosa, bulimia nervosa, etc.*	Eating disorders	305·60, 306·50, 306·58, 306·59	F50	1
**Personality disorders**	Personality disorders	301, 302	F60–F69	10
**Intellectual disability**	Intellectual disability	311·xx, 312·xx, 313·xx, 314·xx, 315·xx	F70 – F79	1
**Pervasive developmental disorders** *Includes autism spectrum disorder*	Developmental disorders	299·00, 299·01, 299·02, 299·03	F84	1
**Childhood autism**	Childhood autism	299.00	F84.0	1
**Behavioral and emotional disorders with onset usually occurring in childhood and adolescence** *Includes attention-deficit hyperactivity disorder, conduct disorders, childhood emotional disorders, etc*	Behavioral disorders	306·x9, 308·0x	F90 – F98	1
**Attention Deficit Hyperactivity Disorder**	ADHD	308.01	F90, F98.8	3
**Oppositional defiant disorder/conduct disorder**	ODD/CD	308.03, 308.04	F90.1, F91	3

ICD-10: International Classification of Diseases, 10th revision (1994-2016); ICD-8: Danish modification of the International Classification of Diseases, 8th revision (1978-1993)

© 2022 He H et al. *JAMA Network Open*.

eTable 3. **Anatomical Therapeutic Chemical (ATC) codes for autoimmune disease and mental disorders medications.**

Medications	ATC
**Autoimmune diseases**	
NSAIDS	M01A
DMARDS	
Azathioprine	L04AX01
Cyclophosphamide	L01AA01
Cyclosporine	L04AD01
Hydroxychloroquine	P01BA02
Leflunomide	L04AA13
Methotrexate	L01BA01, L04AX03
Mycophenolate mofetil	L04AA06
Sulfasalazine	A07EC01
TNF-α antagonists	L04AB
Systemic glucocorticoids	H02AB
Topical glucocorticoids	D07
Antipsoriatics	D05
Tofacitinib	L04AA29
Rituxumab	L01FA01
Pilocarpine	S01EB01
Insulin	A10A
Anticholinesterases	N07AA
Thyroid preparations	H03A
**Mental disorders**	
Psycholeptics	N05
Psychoanaleptics	N06

© 2022 He H et al. *JAMA Network Open.*

eTable 4. **Associations between specific maternal autoimmune diseases and risk of any mental disorder in the offspring by offspring's age**

	Age of offspring at the diagnosis of the mental disorder					
	1-5 years		6-18 years		>18 years	
**Exposures**	No. of events (rate per 1000 person years)	HR (95% CI), adjusted[a]	No. of events (rate per 1000 person years)	HR (95% CI), adjusted[a]	No. of events (rate per 1000 person years)	HR (95% CI), adjusted[a]
**Any autoimmune disease**	994 (4.55)	1.20 (1.13-1.28)	3247 (11.38)	1.16 (1.12-1.20)	1219 (15.57)	1.13 (1.07-1.20)
**Endocrine autoimmune diseases**	362 (4.59)	1.25 (1.13-1.39)	1192 (10.93)	1.17 (1.11-1.24)	586 (15.78)	1.17 (1.08-1.27)
Type 1 Diabetes	181 (4.47)	1.35 (1.17-1.57)	781 (10.95)	1.24 (1.15-1.33)	532 (15.88)	1.19 (1.09-1.30)
Thyreotoxicosis	138 (4.41)	1.08 (0.92-1.28)	364 (11.19)	1.08 (0.98-1.20)	43 (14.57)	0.96 (0.71-1.29)
Autoimmune thyroiditis	41 (5.53)	1.33 (0.98-1.81)	50 (10.21)	1.04 (0.79-1.38)	7 (26.41)	1.84 (0.88-3.86)
Primary adrenocortical insufficiency	10 (8.11)	2.10 (1.13-3.90)	19 (11.74)	1.22 (0.78-1.91)	6 (11.95)	0.86 (0.39-1.92)
**Connective tissue autoimmune diseases**	166 (5.12)	1.28 (1.10-1.49)	488 (12.78)	1.24 (1.13-1.36)	144 (16.97)	1.21 (1.02-1.42)
Rheumatoid arthritis	95 (5.54)	1.42 (1.16-1.74)	233 (11.76)	1.19 (1.05-1.36)	75 (17.09)	1.28 (1.02-1.60)
Juvenile arthritis	57 (6.94)	1.58 (1.22-2.05)	113 (12.01)	0.99 (0.82-1.19)	33 (21.22)	1.27 (0.90-1.78)
Dermatopolymyositis	0	-	10 (9.56)	0.90 (0.48-1.67)	<6 (17.76)	1.36 (0.56-3.26)
Polymyalgia rheumatica/temporal arteritis	<6 (12.94)	2.63 (0.85-8.16)	<6 (30)	2.95 (1.23-7.09)	0	-
Scleroderma	<6 (3.65)	0.90 (0.34-2.39)	22 (17)	1.60 (1.06-2.43)	<6 (12.4)	0.89 (0.33-2.37)
Systemic lupus erythematosus	16 (3.66)	0.96 (0.59-1.57)	86 (15.05)	1.54 (1.25-1.90)	23 (15.7)	1.13 (0.75-1.70)
Sjögren syndrome	<6 (3.76)	0.87 (0.36-2.08)	21 (17.21)	1.59 (1.03-2.43)	<6 (5)	0.39 (0.05-2.75)
Ankylosing Spondylitis	15 (4.56)	1.16 (0.70-1.93)	37 (10.74)	1.11 (0.81-1.54)	15 (17.98)	1.20 (0.72-1.99)
Wegener granulomatosis	<6 (4.06)	1.00 (0.14-7.07)	<6 (11.69)	1.15 (0.37-3.58)	0	-
unexposed	30698 (3)	1.00 (ref)	159229 (8.39)	1.00 (ref)	113165 (12.4)	1.00 (ref)

HR=Hazard Ratio, CI=Confidential Interval

[a]HRs were adjusted for parental psychiatric history, maternal characteristics (parity, age at birth, highest education level, cohabitation with a partner, residence, birth country) and birth characteristics (participant's sex, calendar year of birth).

© 2022 He H et al. *JAMA Network Open.*

eTable 4 (continued). **Associations between specific maternal autoimmune diseases and risk of any mental disorder in the offspring by offspring's age.**

	**Age of offspring at the diagnosis of the mental disorder**					
	**1-5 years**		**6-18 years**		**>18 years**	
**Exposures**	No. of events (rate per 1000 person years)	HR (95% CI), adjusted[a]	No. of events (rate per 1000 person years)	HR (95% CI), adjusted[a]	No. of events (rate per 1000 person years)	HR (95% CI), adjusted[a]
**Gastrointestinal autoimmune diseases**	292 (4.2)	1.10 (0.98-1.24)	962 (11.23)	1.13 (1.06-1.21)	266 (14.32)	1.04 (0.92-1.18)
Celiac disease	26 (4.73)	1.13 (0.77-1.66)	73 (14.17)	1.30 (1.03-1.63)	7 (10.72)	0.64 (0.31-1.35)
Crohn disease	104 (3.98)	1.00 (0.82-1.21)	366 (11.43)	1.07 (0.97-1.19)	106 (17.36)	1.17 (0.97-1.42)
Ulcerative colitis	173 (3.99)	1.08 (0.93-1.26)	558 (10.35)	1.08 (1.00-1.18)	163 (13.2)	1.00 (0.86-1.17)
Primary biliary cirrhosis	<6 (18.87)	4.68 (1.95-11.24)	6 (23)	2.28 (1.03-5.08)	<6 (63.38)	4.18 (0.59-29.68)
Autoimmune hepatitis	19 (8.45)	1.90 (1.21-2.99)	42 (14.87)	1.47 (1.08-1.98)	15 (20.65)	1.26 (0.76-2.10)
**Blood autoimmune diseases**	31 (5)	1.25 (0.88-1.78)	79 (10.55)	1.01 (0.81-1.26)	28 (19.86)	1.26 (0.87-1.82)
Pernicious anemia	15 (8.74)	1.98 (1.20-3.29)	22 (12.08)	1.08 (0.71-1.65)	6 (21.17)	1.24 (0.56-2.76)
Autoimmune hemolytic anemia	<6 (5.82)	1.42 (0.46-4.40)	<6 (6.13)	0.59 (0.22-1.56)	<6 (24.49)	1.51 (0.49-4.69)
Idiopathic thrombocyopenic purpura	13 (3.22)	0.85 (0.49-1.46)	55 (10.76)	1.06 (0.82-1.39)	19 (18.66)	1.22 (0.78-1.91)
**Nervous system autoimmune diseases**	73 (4.38)	1.19 (0.94-1.49)	272 (12.06)	1.23 (1.09-1.38)	89 (14.13)	1.03 (0.84-1.27)
Multiple sclerosis	50 (4.15)	1.11 (0.84-1.47)	192 (12.83)	1.34 (1.16-1.54)	51 (12.86)	0.96 (0.73-1.26)
Guillanian-Barré syndrome	18 (5.23)	1.44 (0.91-2.29)	66 (11.33)	1.09 (0.85-1.38)	32 (18.66)	1.26 (0.89-1.78)
Myasthenia gravis	6 (4.53)	1.31 (0.59-2.92)	15 (7.32)	0.76 (0.46-1.26)	9 (13.38)	1.00 (0.52-1.93)
**Skin autoimmune diseases**	112 (5.34)	1.34 (1.11-1.61)	347 (12.4)	1.16 (1.05-1.29)	119 (16.81)	1.14 (0.95-1.37)
Pemphigus	<6 (6.57)	1.26 (0.31-5.03)	<6 (12.63)	1.07 (0.40-2.86)	<6 (25.31)	1.42 (0.20-10.11)
Pemphigoid	<6 (6.11)	1.11 (0.16-7.86)	<6 (5.32)	0.47 (0.07-3.34)	<6 (68.25)	3.14 (0.79-12.57)
Psoriasis vulgaris	92 (5.66)	1.43 (1.17-1.76)	299 (13.31)	1.23 (1.10-1.38)	100 (16.85)	1.12 (0.92-1.37)
Alopecia areata	9 (3.2)	0.80 (0.41-1.53)	33 (9.67)	0.97 (0.69-1.37)	14 (15.48)	1.20 (0.71-2.03)
Vitiligo	9 (5.64)	1.40 (0.73-2.69)	13 (7.43)	0.76 (0.44-1.30)	<6 (10.61)	0.77 (0.19-3.10)
unexposed	30698 (3)	1.00 (ref)	159229 (8.39)	1.00 (ref)	113165 (12.4)	1.00 (ref)

HR=Hazard Ratio, CI=Confidential Interval

[a]HRs were adjusted for parental psychiatric history, maternal characteristics (parity, age at birth, highest education level, cohabitation with a partner, residence, birth country) and birth characteristics (participant's sex,

© 2022 He H et al. *JAMA Network Open.*

calendar year of birth)

© 2022 He H et al. *JAMA Network Open*.

eTable 5. **Hazard ratios of any mental disorder among the offspring stratified by the numbers of comorbid maternal autoimmune diseases.**

Exposures and Outcomes	No of events	rate per 1000 person years	HR (95% CI), crude	HR (95% CI), adjusted[a]
**Any mental disorder**				
Exposed to one AD	5077	9.37	1.39 (1.35-1.43)	1.16 (1.13-1.20)
Exposed to more than one AD	383	9.50	1.66 (1.51-1.84)	1.16 (1.05-1.28)
Unexposed	303092	7.91	1.00 (ref)	1.00 (ref)
**Organic disorders**				
Exposed to one AD	66	0.11	1.52 (1.20-1.95)	1.50 (1.18-1.92)
Exposed to more than one AD	<6	0.12	2.21 (0.92-5.31)	2.17 (0.90-5.22)
Unexposed	4117	0.10	1.00 (ref)	1.00 (ref)
**Substance use disorders**				
Exposed to one AD	814	3.25	1.10 (1.02-1.17)	1.05 (0.98-1.12)
Exposed to more than one AD	40	2.99	1.15 (0.84-1.56)	1.04 (0.76-1.41)
Unexposed	73465	3.21	1.00 (ref)	1.00 (ref)
**Schizophrenia**				
Exposed to one AD	302	1.19	1.53 (1.36-1.71)	1.35 (1.21-1.51)
Exposed to more than one AD	17	1.25	1.90 (1.18-3.06)	1.42 (0.88-2.28)
Unexposed	20171	0.86	1.00 (ref)	1.00 (ref)
**Mood disorders**				
Exposed to one AD	632	2.50	1.29 (1.19-1.40)	1.14 (1.05-1.23)
Exposed to more than one AD	25	1.85	1.17 (0.79-1.73)	0.87 (0.59-1.29)
Unexposed	51512	2.22	1.00 (ref)	1.00 (ref)
**Neurotic disorders**				
Exposed to one AD	1611	4.00	1.44 (1.37-1.51)	1.21 (1.15-1.27)
Exposed to more than one AD	112	4.28	1.97 (1.64-2.37)	1.34 (1.11-1.61)
unexposed	107385	3.37	1.00 (ref)	1.00 (ref)
**OCD**				
Exposed to one AD	199	0.48	1.81 (1.58-2.09)	1.42 (1.23-1.64)
Exposed to more than one AD	14	0.53	2.26 (1.34-3.81)	1.40 (0.83-2.37)
unexposed	9623	0.30	1.00 (ref)	1.00 (ref)
**Eating disorders**				
Exposed to one AD	244	0.42	1.32 (1.16-1.50)	1.14 (1.00-1.29)
Exposed to more than one AD	13	0.31	1.24 (0.72-2.14)	0.92 (0.53-1.59)
unexposed	16255	0.40	1.00 (ref)	1.00 (ref)

HR=Hazard Ratio, CI=Confidential Interval, AD=autoimmune disease, OCD=Obsessive-Compulsive Disorder, ADHD=Attention Deficit/Hyperactivity Disorder, ODD/CD=oppositional defiant disorder/conduct disorder

[a]HRs were adjusted for parental psychiatric history, maternal characteristics (parity, age at birth, highest education level, cohabitation with a partner, residence, birth country) and birth characteristics (participant's sex, calendar year of birth).

© 2022 He H et al. *JAMA Network Open.*

eTable 5. (Continued) **Hazard ratios of any mental disorder among the offspring stratified by the numbers of comorbid maternal autoimmune diseases.**

Exposures and outcomes	No of events	rate per 1000 person years	HR (95% CI), crude	HR (95% CI), adjusted[a]
**Personality disorders**				
Exposed to one AD	355	1.40	1.22 (1.10-1.35)	1.10 (0.99-1.22)
Exposed to more than one AD	19	1.41	1.52 (0.97-2.38)	1.16 (0.74-1.81)
unexposed	30538	1.31	1.00 (ref)	1.00 (ref)
**Intellectual disability**				
Exposed to one AD	281	0.49	1.25 (1.11-1.41)	1.18 (1.05-1.33)
Exposed to more than one AD	26	0.61	1.44 (0.98-2.11)	1.32 (0.90-1.94)
unexposed	14284	0.35	1.00 (ref)	1.00 (ref)
**Developmental disorders**				
Exposed to one AD	679	1.18	1.67 (1.55-1.80)	1.13 (1.05-1.22)
Exposed to more than one AD	76	1.80	2.48 (1.98-3.10)	1.30 (1.04-1.63)
unexposed	27272	0.67	1.00 (ref)	1.00 (ref)
**Childhood autism**				
Exposed to one AD	270	0.47	1.90 (1.68-2.14)	1.21 (1.07-1.37)
Exposed to more than one AD	29	0.69	2.47 (1.72-3.56)	1.24 (0.86-1.79)
unexposed	8689	0.21	1.00 (ref)	1.00 (ref)
**Behavioral disorders**				
Exposed to one AD	1834	3.25	1.56 (1.49-1.63)	1.20 (1.14-1.25)
Exposed to more than one AD	164	3.95	1.89 (1.62-2.20)	1.18 (1.01-1.37)
unexposed	80984	2.01	1.00 (ref)	1.00 (ref)
**ADHD**				
Exposed to one AD	1001	2.06	1.69 (1.59-1.80)	1.18 (1.11-1.26)
Exposed to more than one AD	98	2.94	2.43 (1.99-2.96)	1.28 (1.05-1.55)
unexposed	43929	1.21	1.00 (ref)	1.00 (ref)
**ODD/CD**				
Exposed to one AD	201	0.41	1.29 (1.12-1.48)	1.12 (0.97-1.29)
Exposed to more than one AD	18	0.53	1.59 (1.00-2.52)	1.20 (0.76-1.91)
unexposed	10766	0.29	1.00 (ref)	1.00 (ref)

HR=Hazard Ratio, CI=Confidential Interval, AD=autoimmune disease, OCD=Obsessive-Compulsive Disorder, ADHD=Attention Deficit/Hyperactivity Disorder, ODD/CD=oppositional defiant disorder/conduct disorder

[a]HRs were adjusted for parental psychiatric history, maternal characteristics (parity, age at birth, highest education level, cohabitation with a partner, residence, birth country) and birth characteristics (participant’s sex, calendar year of birth).

© 2022 He H et al. *JAMA Network Open.*

eTable 6. **Associations between specific maternal autoimmune diseases diagnosed before childbirth and overall mental disorders among term-born individuals (n=2 073 334).**

**Exposures**	**No of events**	**rate per 1000 person years**	**HR (95% CI), crude**	**HR (95% CI), adjusted[a]**
Any autoimmune diseases	4571	9.04	1.38 (1.34-1.42)	1.15 (1.12-1.18)
Endocrine autoimmune diseases	1608	8.99	1.32 (1.25-1.38)	1.16 (1.10-1.22)
Type 1 Diabetes	1039	9.86	1.25 (1.18-1.33)	1.21 (1.14-1.29)
Thyreotoxicosis	474	7.75	1.45 (1.32-1.58)	1.06 (0.96-1.15)
Autoimmune thyroiditis	85	7.34	1.65 (1.33-2.03)	1.16 (0.94-1.43)
Primary adrenocortical insufficiency	33	11.07	1.61 (1.15-2.26)	1.36 (0.97-1.91)
Connective tissue autoimmune diseases	704	9.83	1.58 (1.47-1.70)	1.23 (1.14-1.33)
Rheumatoid arthritis	368	9.74	1.58 (1.42-1.75)	1.28 (1.15-1.41)
Juvenile arthritis	185	10.38	1.73 (1.50-2.00)	1.14 (0.99-1.32)
Dermatopolymyositis	12	6.40	0.94 (0.53-1.66)	0.74 (0.42-1.31)
Polymyalgia rheumatica/temporal arteritis	8	21.97	4.92 (2.41-10.04)	3.35 (1.68-6.70)
Scleroderma	26	10.78	1.76 (1.20-2.58)	1.31 (0.89-1.93)
Systemic lupus erythematosus	102	10.72	1.64 (1.35-1.98)	1.36 (1.12-1.65)
Sjögren syndrome	22	8.91	1.65 (1.09-2.51)	1.20 (0.79-1.82)
Ankylosing Spondylitis	57	8.10	1.32 (1.02-1.71)	1.08 (0.83-1.40)
Wegener granulomatosis	0	-	-	-
Gastrointestinal autoimmune diseases	1347	8.50	1.34 (1.27-1.42)	1.10 (1.05-1.16)
Celiac disease	95	9.13	1.68 (1.38-2.06)	1.17 (0.96-1.43)
Crohn disease	501	8.60	1.38 (1.26-1.50)	1.06 (0.97-1.16)
Ulcerative colitis	799	7.97	1.25 (1.17-1.34)	1.07 (1.00-1.14)
Primary biliary cirrhosis	9	21.35	4.02 (2.10-7.68)	2.92 (1.52-5.61)
Autoimmune hepatitis	64	13.09	2.07 (1.62-2.64)	1.56 (1.22-1.99)
Blood autoimmune diseases	122	8.97	1.46 (1.22-1.74)	1.11 (0.93-1.33)
Pernicious anemia	37	10.49	1.86 (1.35-2.55)	1.27 (0.92-1.76)
Autoimmune hemolytic anemia	9	7.46	1.19 (0.62-2.29)	0.91 (0.47-1.75)
Idiopathic thrombocyopenic purpura	78	8.63	1.37 (1.09-1.71)	1.08 (0.87-1.35)
Nervous system autoimmune diseases	396	9.33	1.38 (1.25-1.52)	1.17 (1.06-1.30)
Multiple sclerosis	271	9.37	1.44 (1.28-1.63)	1.22 (1.09-1.38)
Guillanian-Barré syndrome	101	9.90	1.35 (1.11-1.64)	1.12 (0.92-1.36)
Myasthenia gravis	27	7.25	1.00 (0.68-1.46)	0.92 (0.63-1.34)
Skin autoimmune diseases	505	9.88	1.50 (1.38-1.64)	1.17 (1.07-1.27)
Pemphigus	<6	7.17	1.30 (0.50-3.42)	0.80 (0.30-2.14)
Pemphigoid	<6	10.70	1.81 (0.68-4.77)	1.19 (0.45-3.16)
Psoriasis vulgaris	432	10.61	1.59 (1.45-1.75)	1.23 (1.12-1.35)
Alopecia areata	48	7.32	1.16 (0.87-1.53)	0.93 (0.70-1.24)
Vitiligo	20	6.26	1.12 (0.73-1.74)	0.86 (0.56-1.33)

HR=Hazard Ratio, CI=Confidential Interval

[a]HRs were adjusted for parental psychiatric history, maternal characteristics (parity, age at birth, highest education level, cohabitation with a partner, residence, birth country) and birth characteristics (participant's sex, calendar year of birth).

© 2022 He H et al. *JAMA Network Open.*

eTable 7. **Hazard ratios of specific mental disorders according to prenatal exposure to any maternal autoimmune disease among term-born individuals (n=2 073 334).**

Exposures and outcomes	No of events	rate per 1000 person years	HR (95% CI), crude	HR (95% CI), adjusted[a]
**Any mental disorder**				
exposed	4571	9.04	1.38 (1.34-1.42)	1.15 (1.12-1.18)
unexposed	273780	7.88	1.00 (ref)	1.00 (ref)
**Organic disorders**				
exposed	56	0.10	1.51 (1.16-1.96)	1.50 (1.15-1.95)
unexposed	3532	0.10	1.00 (ref)	1.00 (ref)
**Substance use disorders**				
exposed	720	3.21	1.09 (1.01-1.17)	1.05 (0.97-1.12)
unexposed	66169	3.23	1.00 (ref)	1.00 (ref)
**Schizophrenia**				
exposed	265	1.16	1.51 (1.34-1.71)	1.34 (1.19-1.52)
unexposed	18081	0.86	1.00 (ref)	1.00 (ref)
**Mood disorders**				
exposed	547	2.41	1.25 (1.15-1.36)	1.11 (1.02-1.21)
unexposed	46266	2.23	1.00 (ref)	1.00 (ref)
**Neurotic disorders**				
exposed	1423	3.85	1.41 (1.34-1.49)	1.19 (1.13-1.25)
unexposed	96928	3.37	1.00 (ref)	1.00 (ref)
**OCD**				
exposed	182	0.48	1.80 (1.55-2.09)	1.41 (1.22-1.64)
unexposed	8842	0.30	1.00 (ref)	1.00 (ref)
**Eating disorders**				
exposed	206	0.38	1.23 (1.07-1.41)	1.07 (0.93-1.22)
unexposed	14825	0.40	1.00 (ref)	1.00 (ref)
**Personality disorders**				
exposed	317	1.39	1.23 (1.10-1.37)	1.12 (1.00-1.25)
unexposed	27380	1.31	1.00 (ref)	1.00 (ref)
**Intellectual disability**				
exposed	234	0.44	1.20 (1.05-1.36)	1.13 (1.00-1.29)
unexposed	12177	0.33	1.00 (ref)	1.00 (ref)
**Developmental disorders**				
exposed	657	1.23	1.74 (1.61-1.87)	1.16 (1.08-1.26)
unexposed	24936	0.68	1.00 (ref)	1.00 (ref)
**Childhood autism**				
exposed	254	0.47	1.92 (1.69-2.17)	1.21 (1.07-1.37)
unexposed	7852	0.21	1.00 (ref)	1.00 (ref)
**Behavioral disorders**				
exposed	1651	3.14	1.51 (1.44-1.59)	1.16 (1.11-1.22)
unexposed	73735	2.02	1.00 (ref)	1.00 (ref)
**ADHD**				
exposed	905	2.02	1.66 (1.55-1.77)	1.16 (1.08-1.23)
unexposed	40012	1.22	1.00 (ref)	1.00 (ref)
**ODD/CD**				
exposed	175	0.39	1.21 (1.05-1.41)	1.07 (0.92-1.24)
unexposed	9793	0.30	1.00 (ref)	1.00 (ref)

HR=Hazard Ratio, CI=Confidential Interval, OCD=Obsessive-Compulsive Disorder, ADHD=Attention Deficit/Hyperactivity Disorder, ODD/CD=oppositional defiant disorder/conduct disorder.

[a]HRs were adjusted for parental psychiatric history, maternal characteristics (parity, age at birth, highest education level, cohabitation with a partner, residence, birth country) and birth characteristics (participant's sex, calendar year of birth).

© 2022 He H et al. *JAMA Network Open.*

eTable 8. **Associations between specific maternal autoimmune diseases diagnosed before child birth and overall mental disorders among individuals without congenital malformations of the nervous system or chromosomal abnormalities.**

**Exposures**	**No of events**	**rate per 1000 person years**	**HR (95% CI), crude**	**HR (95% CI), adjusted[a]**
Any autoimmune diseases	5362	9.27	1.41 (1.38-1.45)	1.17 (1.13-1.20)
Endocrine autoimmune diseases	2103	9.40	1.37 (1.32-1.43)	1.19 (1.14-1.24)
Type 1 Diabetes	1475	10.23	1.33 (1.26-1.40)	1.24 (1.18-1.31)
Thyreotoxicosis	532	8.00	1.51 (1.38-1.64)	1.08 (0.99-1.17)
Autoimmune thyroiditis	91	7.28	1.63 (1.33-2.01)	1.13 (0.92-1.39)
Primary adrenocortical insufficiency	35	10.44	1.54 (1.10-2.14)	1.29 (0.92-1.79)
Connective tissue autoimmune diseases	784	9.97	1.61 (1.50-1.72)	1.25 (1.16-1.34)
Rheumatoid arthritis	396	9.65	1.58 (1.43-1.74)	1.26 (1.15-1.39)
Juvenile arthritis	198	10.39	1.73 (1.51-1.99)	1.14 (1.00-1.32)
Dermatopolymyositis	15	7.30	1.09 (0.66-1.81)	0.83 (0.50-1.38)
Polymyalgia rheumatica/temporal arteritis	7	17.82	4.46 (2.16-9.23)	2.56 (1.22-5.36)
Scleroderma	29	10.72	1.76 (1.23-2.53)	1.31 (0.91-1.89)
Systemic lupus erythematosus	124	10.81	1.67 (1.40-1.99)	1.36 (1.14-1.62)
Sjögren syndrome	27	9.95	1.88 (1.30-2.73)	1.31 (0.90-1.91)
Ankylosing Spondylitis	67	8.87	1.46 (1.15-1.85)	1.17 (0.92-1.49)
Wegener granulomatosis	<6	7.50	1.32 (0.50-3.46)	0.96 (0.36-2.57)
Gastrointestinal autoimmune diseases	1487	8.61	1.38 (1.31-1.45)	1.11 (1.05-1.17)
Celiac disease	102	9.07	1.70 (1.40-2.06)	1.15 (0.94-1.39)
Crohn disease	567	8.87	1.44 (1.32-1.56)	1.09 (1.00-1.18)
Ulcerative colitis	873	8.01	1.27 (1.19-1.36)	1.07 (1.00-1.14)
Primary biliary cirrhosis	12	22.15	4.41 (2.53-7.68)	3.13 (1.78-5.52)
Autoimmune hepatitis	75	12.96	2.03 (1.62-2.55)	1.51 (1.20-1.89)
Blood autoimmune diseases	136	9.06	1.48 (1.25-1.75)	1.11 (0.94-1.32)
Pernicious anemia	42	11.03	1.91 (1.41-2.59)	1.31 (0.97-1.77)
Autoimmune hemolytic anemia	10	7.89	1.32 (0.72-2.44)	0.95 (0.51-1.76)
Idiopathic thrombocyopenic purpura	86	8.50	1.35 (1.10-1.67)	1.06 (0.86-1.31)
Nervous system autoimmune diseases	426	9.39	1.41 (1.28-1.55)	1.17 (1.07-1.29)
Multiple sclerosis	288	9.34	1.46 (1.30-1.64)	1.22 (1.08-1.37)
Guillanian-Barré syndrome	114	10.39	1.43 (1.19-1.72)	1.16 (0.97-1.40)
Myasthenia gravis	29	7.20	1.00 (0.69-1.44)	0.88 (0.61-1.27)
Skin autoimmune diseases	572	10.26	1.58 (1.46-1.71)	1.20 (1.11-1.30)
Pemphigus	6	9.41	1.70 (0.77-3.76)	1.04 (0.47-2.32)
Pemphigoid	<6	11.04	1.96 (0.74-5.13)	1.15 (0.43-3.08)
Psoriasis vulgaris	486	10.93	1.64 (1.50-1.80)	1.25 (1.14-1.37)
Alopecia areata	56	7.90	1.28 (0.99-1.66)	1.02 (0.78-1.32)
Vitiligo	24	6.84	1.27 (0.85-1.88)	0.94 (0.63-1.40)

HR=Hazard Ratio, CI=Confidential Interval

[a]HRs were adjusted for parental psychiatric history, maternal characteristics (parity, age at birth, highest education level, cohabitation with a partner, residence, birth country) and birth characteristics (participant’s sex, calendar year of birth).

© 2022 He H et al. *JAMA Network Open.*

eTable 9. **Hazard ratios of specific mental disorder according to prenatal exposure to any maternal autoimmune disease among individuals without congenital malformations of the nervous system and chromosomal abnormalities.**

Exposures and outcomes	No of events	rate per 1000 person years	HR (95% CI), crude	HR (95% CI), adjusted[a]
**Any mental disorder**				
exposed	5362	9.27	1.41 (1.38-1.45)	1.17 (1.13-1.20)
unexposed	298527	7.83	1.00 (ref)	1.00 (ref)
**Organic disorders**				
exposed	70	0.11	1.59 (1.26-2.02)	1.57 (1.24-1.99)
unexposed	3979	0.10	1.00 (ref)	1.00 (ref)
**Substance use disorders**				
exposed	848	3.24	1.10 (1.03-1.17)	1.05 (0.98-1.12)
unexposed	73101	3.22	1.00 (ref)	1.00 (ref)
**Schizophrenia**				
exposed	318	1.19	1.55 (1.39-1.73)	1.36 (1.22-1.52)
unexposed	20008	0.86	1.00 (ref)	1.00 (ref)
**Mood disorders**				
exposed	655	2.48	1.29 (1.20-1.40)	1.13 (1.04-1.22)
unexposed	51185	2.22	1.00 (ref)	1.00 (ref)
**Neurotic disorders**				
exposed	1704	4.00	1.46 (1.40-1.54)	1.21 (1.15-1.27)
unexposed	106562	3.36	1.00 (ref)	1.00 (ref)
**OCD**				
exposed	209	0.48	1.82 (1.58-2.08)	1.41 (1.22-1.61)
unexposed	9557	0.30	1.00 (ref)	1.00 (ref)
**Eating disorders**				
exposed	250	0.41	1.30 (1.15-1.47)	1.11 (0.98-1.26)
unexposed	16085	0.40	1.00 (ref)	1.00 (ref)
**Personality disorders**				
exposed	372	1.40	1.23 (1.11-1.37)	1.10 (1.00-1.22)
unexposed	30350	1.31	1.00 (ref)	1.00 (ref)
**Intellectual disability**				
exposed	272	0.44	1.38 (1.22-1.55)	1.28 (1.13-1.44)
unexposed	11906	0.29	1.00 (ref)	1.00 (ref)
**Developmental disorders**				
exposed	738	1.21	1.74 (1.62-1.87)	1.15 (1.07-1.24)
unexposed	26563	0.66	1.00 (ref)	1.00 (ref)
**Childhood autism**				
exposed	287	0.47	1.94 (1.73-2.18)	1.21 (1.08-1.37)
unexposed	8344	0.21	1.00 (ref)	1.00 (ref)
**Behavioral disorders**				
exposed	1972	3.27	1.58 (1.51-1.65)	1.19 (1.14-1.25)
unexposed	80048	2.00	1.00 (ref)	1.00 (ref)
**ADHD**				
exposed	1083	2.10	1.73 (1.63-1.84)	1.19 (1.12-1.26)
unexposed	43466	1.20	1.00 (ref)	1.00 (ref)
**ODD/CD**				
exposed	216	0.42	1.31 (1.14-1.50)	1.12 (0.98-1.29)
unexposed	10638	0.29	1.00 (ref)	1.00 (ref)

HR=Hazard Ratio, CI=Confidential Interval, OCD=Obsessive-Compulsive Disorder, ADHD=Attention Deficit/Hyperactivity Disorder, ODD/CD=oppositional defiant disorder/conduct disorder.

[a]HRs were adjusted for parental psychiatric history, maternal characteristics (parity, age at birth, highest education level, cohabitation with a partner, residence, birth country) and birth characteristics (participant's sex, calendar year of birth).

© 2022 He H et al. *JAMA Network Open.*

eTable 10. **Associations between specific autoimmune diseases of mothers and overall mental disorders in the offspring born during 1998-2007.**

Exposures	No of events	rate per 1000 person years	HR (95% CI), crude	HR (95% CI), adjusted[a]
Any autoimmune diseases	2045	9.88	1.26 (1.21-1.32)	1.19 (1.14-1.25)
Endocrine autoimmune diseases	705	9.90	1.27 (1.18-1.37)	1.21 (1.12-1.30)
Type 1 Diabetes	327	11.48	1.43 (1.29-1.60)	1.28 (1.15-1.43)
Thyreotoxicosis	333	8.97	1.17 (1.05-1.31)	1.16 (1.04-1.29)
Autoimmune thyroiditis	51	8.45	1.13 (0.86-1.48)	1.20 (0.91-1.58)
Primary adrenocortical insufficiency	8	9.71	1.30 (0.65-2.59)	1.12 (0.56-2.24)
Connective tissue autoimmune diseases	327	10.68	1.38 (1.23-1.53)	1.28 (1.15-1.42)
Rheumatoid arthritis	169	10.31	1.34 (1.15-1.55)	1.29 (1.11-1.50)
Juvenile arthritis	85	10.50	1.34 (1.08-1.66)	1.11 (0.90-1.37)
Dermatopolymyositis	<6	4.10	0.52 (0.17-1.60)	0.45 (0.15-1.41)
Polymyalgia rheumatica/temporal arteritis	<6	13.29	1.88 (0.61-5.75)	1.85 (0.60-5.74)
Scleroderma	16	14.61	1.89 (1.16-3.09)	1.69 (1.03-2.76)
Systemic lupus erythematosus	47	11.74	1.52 (1.15-2.03)	1.45 (1.09-1.93)
Sjögren syndrome	15	11.68	1.51 (0.91-2.49)	1.39 (0.84-2.30)
Ankylosing Spondylitis	24	9.20	1.19 (0.80-1.78)	1.16 (0.78-1.73)
Wegener granulomatosis	<6	9.04	1.16 (0.29-4.60)	1.17 (0.29-4.66)
Gastrointestinal autoimmune diseases	640	9.32	1.19 (1.10-1.29)	1.14 (1.06-1.24)
Celiac disease	53	10.62	1.40 (1.07-1.83)	1.30 (1.00-1.71)
Crohn disease	239	9.13	1.16 (1.02-1.32)	1.05 (0.92-1.19)
Ulcerative colitis	377	8.80	1.13 (1.02-1.25)	1.12 (1.01-1.24)
Primary biliary cirrhosis	<6	19.45	2.52 (1.05-6.04)	2.52 (1.05-6.04)
Autoimmune hepatitis	31	13.98	1.81 (1.27-2.57)	1.82 (1.28-2.59)
Blood autoimmune diseases	64	10.04	1.29 (1.01-1.65)	1.22 (0.96-1.56)
Pernicious anemia	21	12.07	1.58 (1.03-2.42)	1.45 (0.94-2.22)
Autoimmune hemolytic anemia	<6	8.57	1.10 (0.46-2.65)	0.98 (0.41-2.36)
Idiopathic thrombocyopenic purpura	39	9.43	1.20 (0.88-1.65)	1.17 (0.85-1.60)
Nervous system autoimmune diseases	163	10.63	1.37 (1.17-1.59)	1.27 (1.09-1.48)
Multiple sclerosis	125	11.19	1.46 (1.23-1.74)	1.38 (1.16-1.65)
Guillanian-Barré syndrome	30	10.21	1.26 (0.88-1.80)	1.07 (0.75-1.53)
Myasthenia gravis	10	7.27	0.91 (0.49-1.69)	0.89 (0.48-1.66)
Skin autoimmune diseases	224	10.76	1.37 (1.20-1.56)	1.22 (1.07-1.39)
Pemphigus	<6	10.42	1.36 (0.44-4.23)	1.11 (0.36-3.43)
Pemphigoid	<6	11.41	1.48 (0.37-5.90)	1.32 (0.33-5.26)
Psoriasis vulgaris	188	11.89	1.52 (1.31-1.75)	1.31 (1.14-1.51)
Alopecia areata	23	8.11	1.02 (0.68-1.54)	0.96 (0.64-1.44)
Vitiligo	12	6.46	0.85 (0.48-1.48)	0.87 (0.50-1.54)

HR=Hazard Ratio, CI=Confidential Interval

[a]HRs were adjusted for parental psychiatric history, maternal characteristics (parity, age at birth, highest education level,

© 2022 He H et al. *JAMA Network Open.*

cohabitation with a partner, residence, birth country) and birth characteristics (participant's sex, calendar year of birth).

© 2022 He H et al. *JAMA Network Open*.

eTable 11. **Associations between specific autoimmune diseases of mothers and overall mental disorders in the offspring born during 2008-2015.**

Exposures	No of events	rate per 1000 person years	HR (95% CI), crude	HR (95% CI), adjusted[a]
Any autoimmune diseases	517	6.58	1.32 (1.21-1.44)	1.30 (1.19-1.42)
Endocrine autoimmune diseases	171	6.56	1.31 (1.13-1.52)	1.31 (1.13-1.53)
Type 1 Diabetes	67	8.12	1.62 (1.27-2.05)	1.48 (1.17-1.89)
Thyreotoxicosis	72	5.33	1.04 (0.83-1.32)	1.10 (0.87-1.38)
Autoimmune thyroiditis	31	6.34	1.34 (0.94-1.90)	1.41 (0.99-2.00)
Primary adrenocortical insufficiency	9	18.69	3.76 (1.96-7.23)	3.85 (2.00-7.40)
Connective tissue autoimmune diseases	105	7.82	1.58 (1.30-1.91)	1.53 (1.26-1.85)
Rheumatoid arthritis	58	8.02	1.60 (1.24-2.08)	1.59 (1.23-2.06)
Juvenile arthritis	40	11.64	2.37 (1.74-3.24)	2.08 (1.53-2.84)
Dermatopolymyositis	0	-	-	-
Polymyalgia rheumatica/temporal arteritis	<6	14.81	3.08 (0.78-12.1)	2.86 (0.72-11.4)
Scleroderma	<6	4.29	0.83 (0.21-3.29)	0.82 (0.20-3.26)
Systemic lupus erythematosus	7	4.59	0.93 (0.44-1.94)	0.91 (0.43-1.91)
Sjögren syndrome	<6	7.33	1.54 (0.64-3.70)	1.49 (0.62-3.58)
Ankylosing Spondylitis	11	6.60	1.38 (0.77-2.50)	1.37 (0.76-2.48)
Wegener granulomatosis	<6	16.70	3.37 (0.85-13.4)	3.47 (0.87-13.8)
Gastrointestinal autoimmune diseases	162	5.97	1.20 (1.02-1.40)	1.17 (1.00-1.37)
Celiac disease	19	6.70	1.39 (0.89-2.18)	1.35 (0.86-2.12)
Crohn disease	60	5.82	1.16 (0.90-1.50)	1.11 (0.86-1.44)
Ulcerative colitis	89	5.27	1.05 (0.85-1.29)	1.06 (0.86-1.30)
Primary biliary cirrhosis	<6	15.68	3.11 (0.79-12.2)	3.13 (0.78-12.5)
Autoimmune hepatitis	9	11.71	2.35 (1.22-4.51)	1.97 (1.02-3.79)
Blood autoimmune diseases	8	3.35	0.67 (0.34-1.34)	0.68 (0.34-1.36)
Pernicious anemia	<6	6.46	1.28 (0.53-3.06)	1.27 (0.53-3.04)
Autoimmune hemolytic anemia	<6	5.70	1.14 (0.16-8.02)	1.34 (0.19-9.50)
Idiopathic thrombocyopenic purpura	<6	1.37	0.28 (0.07-1.11)	0.28 (0.07-1.11)
Nervous system autoimmune diseases	43	7.59	1.53 (1.13-2.06)	1.54 (1.14-2.08)
Multiple sclerosis	30	6.48	1.30 (0.91-1.86)	1.33 (0.93-1.90)
Guillanian-Barré syndrome	9	12.44	2.46 (1.28-4.71)	2.36 (1.23-4.54)
Myasthenia gravis	<6	11.97	2.46 (0.93-6.52)	2.42 (0.91-6.45)
Skin autoimmune diseases	54	7.58	1.52 (1.16-1.98)	1.41 (1.08-1.85)
Pemphigus	<6	7.43	1.55 (0.22-10.92)	1.03 (0.15-7.34)
Pemphigoid	0	-	-	-
Psoriasis vulgaris	46	8.88	1.77 (1.33-2.37)	1.64 (1.23-2.20)
Alopecia areata	<6	2.63	0.54 (0.17-1.65)	0.52 (0.17-1.61)
Vitiligo	<6	5.95	1.20 (0.45-3.17)	1.15 (0.43-3.06)

HR=Hazard Ratio, CI=Confidential Interval

[a]HRs were adjusted for parental psychiatric history, maternal characteristics (parity, age at birth, highest education level,

cohabitation with a partner, residence, birth country) and birth characteristics (participant's sex, calendar year of birth).

© 2022 He H et al. *JAMA Network Open*.

eTable 12. **Associations between specific paternal autoimmune diseases diagnosed before childbirth and overall mental disorders in offspring.**

**Exposures**	**No of events**	**rate per 1000 person years**	**HR (95% CI), crude**	**HR (95% CI), adjusted[a]**
Any autoimmune diseases	3903	8.84	1.30 (1.26-1.34)	1.09 (1.05-1.12)
Endocrine autoimmune diseases	1590	9.36	1.30 (1.24-1.37)	1.12 (1.06-1.17)
Type 1 Diabetes	1475	9.45	1.29 (1.23-1.36)	1.12 (1.06-1.18)
Thyreotoxicosis	93	8.63	1.55 (1.26-1.89)	1.16 (0.95-1.42)
Autoimmune thyroiditis	9	6.23	1.22 (0.64-2.32)	0.92 (0.48-1.78)
Primary adrenocortical insufficiency	21	8.17	1.19 (0.78-1.81)	0.92 (0.60-1.41)
Connective tissue autoimmune diseases	497	8.51	1.31 (1.20-1.43)	1.06 (0.97-1.16)
Rheumatoid arthritis	205	8.79	1.32 (1.15-1.52)	1.11 (0.96-1.27)
Juvenile arthritis	78	8.37	1.42 (1.14-1.78)	0.97 (0.77-1.21)
Dermatopolymyositis	21	10.96	1.68 (1.10-2.57)	1.28 (0.83-1.96)
Polymyalgia rheumatica/temporal arteritis	<6	4.42	0.79 (0.26-2.39)	0.48 (0.15-1.49)
Scleroderma	6	5.08	0.77 (0.35-1.69)	0.59 (0.27-1.32)
Systemic lupus erythematosus	17	9.57	1.47 (0.91-2.36)	1.31 (0.82-2.11)
Sjögren syndrome	8	7.73	1.27 (0.64-2.52)	1.00 (0.50-1.99)
Ankylosing Spondylitis	195	8.64	1.32 (1.14-1.52)	1.11 (0.96-1.28)
Wegener granulomatosis	<6	6.53	1.00 (0.37-2.73)	1.05 (0.39-2.80)
Gastrointestinal autoimmune diseases	1093	8.02	1.26 (1.18-1.33)	1.02 (0.96-1.09)
Celiac disease	60	10.19	1.89 (1.47-2.43)	1.17 (0.91-1.51)
Crohn disease	366	8.04	1.29 (1.16-1.42)	1.01 (0.91-1.12)
Ulcerative colitis	686	7.77	1.23 (1.14-1.33)	1.04 (0.96-1.12)
Primary biliary cirrhosis	<6	2.40	0.37 (0.06-2.43)	0.36 (0.05-2.57)
Autoimmune hepatitis	79	9.54	1.29 (1.03-1.61)	0.98 (0.79-1.23)
Blood autoimmune diseases	62	8.44	1.34 (1.04-1.72)	1.10 (0.86-1.42)
Pernicious anemia	17	10.86	1.58 (0.98-2.54)	1.40 (0.87-2.25)
Autoimmune hemolytic anemia	8	9.45	1.61 (0.81-3.21)	1.28 (0.64-2.55)
Idiopathic thrombocyopenic purpura	38	7.61	1.26 (0.92-1.73)	1.00 (0.73-1.37)
Nervous system autoimmune diseases	315	9.07	1.28 (1.14-1.43)	1.10 (0.99-1.23)
Multiple sclerosis	171	8.23	1.19 (1.02-1.38)	1.02 (0.88-1.19)
Guillanian-Barré syndrome	127	10.28	1.42 (1.20-1.69)	1.19 (1.00-1.42)
Myasthenia gravis	22	11.30	1.62 (1.07-2.46)	1.47 (0.97-2.23)
Skin autoimmune diseases	406	9.64	1.49 (1.35-1.64)	1.16 (1.05-1.28)
Pemphigus	<6	2.62	0.60 (0.10-3.52)	0.32 (0.05-2.27)
Pemphigoid	0	-	-	-
Psoriasis vulgaris	366	10.14	1.52 (1.37-1.68)	1.20 (1.08-1.33)
Alopecia areata	21	6.32	1.25 (0.82-1.91)	0.81 (0.53-1.24)
Vitiligo	18	7.34	1.38 (0.87-2.19)	1.06 (0.67-1.69)

HR=Hazard Ratio, CI=Confidential Interval

[a]HRs were adjusted for parental psychiatric history, maternal characteristics (parity, age at birth, highest education level,

© 2022 He H et al. *JAMA Network Open.*

cohabitation with a partner, residence, birth country) and birth characteristics (participant's sex, calendar year of birth).

© 2022 He H et al. *JAMA Network Open*.

eTable 13. **Hazard ratios of specific mental disorder in offspring according to prenatal exposure to any paternal autoimmune disease diagnosed before childbirth.**

Exposures and outcomes	No of events	rate per 1000 person years	HR (95% CI), crude	HR (95% CI), adjusted[a]
**Any mental disorder**				
exposed	3903	8.84	1.30 (1.26-1.34)	1.09 (1.05-1.12)
unexposed	303163	7.91	1.00 (ref)	1.00 (ref)
**Organic disorders**				
exposed	52	0.11	1.50 (1.14-1.97)	1.47 (1.12-1.93)
unexposed	4099	0.10	1.00 (ref)	1.00 (ref)
**Substance use disorders**				
exposed	678	3.32	1.12 (1.04-1.21)	1.06 (0.98-1.15)
unexposed	73125	3.21	1.00 (ref)	1.00 (ref)
**Schizophrenia**				
exposed	221	1.06	1.37 (1.20-1.57)	1.16 (1.02-1.33)
unexposed	20091	0.86	1.00 (ref)	1.00 (ref)
**Mood disorders**				
exposed	500	2.42	1.27 (1.16-1.39)	1.09 (1.00-1.19)
unexposed	51369	2.22	1.00 (ref)	1.00 (ref)
**Neurotic disorders**				
exposed	1251	3.79	1.37 (1.30-1.45)	1.13 (1.07-1.19)
unexposed	107264	3.37	1.00 (ref)	1.00 (ref)
**OCD**				
exposed	138	0.41	1.52 (1.29-1.80)	1.20 (1.01-1.42)
unexposed	9671	0.30	1.00 (ref)	1.00 (ref)
**Eating disorders**				
exposed	207	0.44	1.35 (1.18-1.55)	1.17 (1.02-1.34)
unexposed	16246	0.40	1.00 (ref)	1.00 (ref)
**Personality disorders**				
exposed	290	1.39	1.23 (1.10-1.39)	1.10 (0.98-1.23)
unexposed	30381	1.31	1.00 (ref)	1.00 (ref)
**Intellectual disability**				
exposed	186	0.40	1.01 (0.88-1.17)	0.96 (0.83-1.10)
unexposed	14337	0.35	1.00 (ref)	1.00 (ref)
**Developmental disorders**				
exposed	508	1.09	1.51 (1.39-1.65)	1.05 (0.96-1.14)
unexposed	27429	0.67	1.00 (ref)	1.00 (ref)
**Childhood autism**				
exposed	199	0.42	1.69 (1.47-1.95)	1.12 (0.97-1.28)
unexposed	8773	0.21	1.00 (ref)	1.00 (ref)
**Behavioral disorders**				
exposed	1364	2.96	1.41 (1.33-1.48)	1.08 (1.03-1.14)
unexposed	81273	2.02	1.00 (ref)	1.00 (ref)
**ADHD**				
exposed	763	1.93	1.56 (1.46-1.68)	1.09 (1.02-1.17)
unexposed	44091	1.21	1.00 (ref)	1.00 (ref)
**ODD/CD**				
exposed	166	0.42	1.29 (1.11-1.51)	1.11 (0.96-1.30)
unexposed	10756	0.29	1.00 (ref)	1.00 (ref)

HR=Hazard Ratio, CI=Confidential Interval, OCD=Obsessive-Compulsive Disorder, ADHD=Attention Deficit/Hyperactivity Disorder, ODD/CD=oppositional defiant disorder/conduct disorder.

[a]HRs were adjusted for parental psychiatric history, maternal characteristics (parity, age at birth, highest education level, cohabitation with a partner, residence, birth country) and birth characteristics (participant's sex, calendar year of birth).

© 2022 He H et al. *JAMA Network Open.*

eTable 14. **Associations between specific maternal autoimmune diseases diagnosed before childbirth and overall mental disorders, adjusted for medications used by mothers before childbirth in addition to the variables in the main model.**

Exposures	No of events/rate per 1000 person years	HR (95% CI), crude	HR (95% CI), Model 1	HR (95% CI), Model 2	HR (95% CI), Model 3
Any autoimmune diseases	2562/8.97	1.28(1.23-1.33)	1.21(1.17-1.26)	1.18(1.13-1.22)	1.16(1.11-1.20)
Endocrine autoimmune diseases	876/9.01	1.29(1.20-1.38)	1.23(1.15-1.32)	1.19(1.11-1.27)	1.18(1.10-1.26)
Type 1 Diabetes	394/10.73	1.47(1.33-1.62)	1.31(1.19-1.45)	1.25(1.13-1.38)	1.24(1.13-1.37)
Thyreotoxicosis	405/8	1.15(1.05-1.27)	1.15(1.04-1.27)	1.12(1.02-1.24)	1.11(1.00-1.22)
Autoimmune thyroiditis	82/7.51	1.22(0.98-1.52)	1.27(1.02-1.57)	1.22(0.98-1.51)	1.20(0.97-1.49)
Primary adrenocortical insufficiency	17/13.02	2.02(1.26-3.25)	1.78(1.11-2.87)	1.73(1.08-2.78)	1.69(1.05-2.72)
Connective tissue autoimmune diseases	432/9.81	1.44(1.31-1.58)	1.33(1.21-1.46)	1.28(1.17-1.41)	1.26(1.15-1.39)
Rheumatoid arthritis	227/9.61	1.41(1.24-1.61)	1.35(1.19-1.54)	1.30(1.14-1.48)	1.28(1.13-1.46)
Juvenile arthritis	125/10.84	1.58(1.33-1.88)	1.30(1.09-1.55)	1.27(1.07-1.51)	1.27(1.06-1.51)
Dermatopolymyositis	<6/3.12	0.45(0.15-1.35)	0.38(0.12-1.18)	0.37(0.12-1.14)	0.35(0.11-1.08)
Polymyalgia rheumatica/temporal arteritis	<6/13.86	2.22(0.93-5.33)	2.11(0.88-5.07)	2.04(0.85-4.89)	1.89(0.79-4.54)
Scleroderma	18/11.53	1.68(1.06-2.67)	1.51(0.95-2.40)	1.49(0.94-2.36)	1.43(0.90-2.27)
Systemic lupus erythematosus	54/9.77	1.41(1.08-1.84)	1.35(1.04-1.77)	1.30(0.99-1.69)	1.27(0.97-1.66)
Sjögren syndrome	20/10.17	1.54(0.99-2.38)	1.42(0.91-2.20)	1.36(0.88-2.11)	1.33(0.86-2.07)
Ankylosing Spondylitis	35/8.19	1.26(0.90-1.75)	1.22(0.87-1.70)	1.17(0.84-1.63)	1.14(0.82-1.59)
Wegener granulomatosis	<6/11.73	1.76(0.67-4.66)	1.70(0.64-4.54)	1.63(0.61-4.34)	1.58(0.59-4.20)
Gastrointestinal autoimmune diseases	802/8.37	1.20(1.12-1.29)	1.15(1.07-1.23)	1.12(1.04-1.20)	1.10(1.02-1.18)
Celiac disease	72/9.2	1.42(1.13-1.79)	1.31(1.04-1.65)	1.29(1.02-1.62)	1.27(1.01-1.60)
Crohn disease	299/8.2	1.17(1.04-1.31)	1.06(0.95-1.19)	1.03(0.92-1.15)	1.01(0.90-1.13)
Ulcerative colitis	466/7.8	1.12(1.02-1.23)	1.10(1.01-1.21)	1.08(0.98-1.18)	1.06(0.96-1.16)
Primary biliary cirrhosis	7/18.2	2.71(1.30-5.66)	2.73(1.31-5.71)	2.62(1.25-5.47)	2.46(1.18-5.13)
Autoimmune hepatitis	40/13.4	1.92(1.41-2.61)	1.87(1.37-2.55)	1.83(1.34-2.49)	1.75(1.29-2.39)
Blood autoimmune diseases	72/8.22	1.18(0.93-1.48)	1.12(0.89-1.41)	1.10(0.87-1.38)	1.10(0.87-1.38)
Pernicious anemia	26/10.35	1.53(1.04-2.24)	1.40(0.95-2.05)	1.37(0.93-2.01)	1.37(0.94-2.02)
Autoimmune hemolytic anemia	6/7.9	1.11(0.50-2.47)	1.01(0.45-2.25)	0.98(0.44-2.18)	0.96(0.43-2.15)
Idiopathic thrombocyopenic purpura	41/7.33	1.04(0.77-1.41)	1.01(0.74-1.37)	0.99(0.73-1.35)	0.99(0.73-1.35)
Nervous system autoimmune diseases	206/9.81	1.41(1.23-1.61)	1.32(1.15-1.51)	1.29(1.13-1.48)	1.24(1.08-1.42)
Multiple sclerosis	155/9.81	1.44(1.23-1.68)	1.37(1.17-1.61)	1.35(1.15-1.58)	1.28(1.09-1.50)
Guillanian-Barré syndrome	39/10.65	1.42(1.04-1.94)	1.22(0.89-1.67)	1.21(0.88-1.65)	1.17(0.85-1.60)
Myasthenia gravis	14/8.19	1.11(0.66-1.87)	1.09(0.64-1.83)	1.04(0.62-1.76)	1.05(0.62-1.77)
Skin autoimmune diseases	278/9.95	1.40(1.25-1.58)	1.25(1.11-1.41)	1.21(1.07-1.36)	1.19(1.06-1.34)
Pemphigus	<6/9.47	1.42(0.53-3.79)	1.06(0.40-2.83)	1.04(0.39-2.78)	1.00(0.37-2.66)
Pemphigoid	<6/8.2	1.22(0.31-4.81)	0.95(0.24-3.79)	0.93(0.23-3.70)	0.88(0.22-3.50)
Psoriasis vulgaris	234/11.15	1.56(1.37-1.78)	1.37(1.20-1.56)	1.31(1.16-1.49)	1.30(1.14-1.47)
Alopecia areata	26/6.54	0.93(0.64-1.37)	0.88(0.60-1.30)	0.85(0.58-1.24)	0.85(0.58-1.25)
Vitiligo	16/6.32	0.91(0.56-1.48)	0.93(0.57-1.52)	0.91(0.56-1.49)	0.91(0.56-1.49)

HR=Hazard Ratio, CI=Confidential Interval

Model 1: adjusted for parental psychiatric history, maternal characteristics (parity, age at birth, highest education level, cohabitation with a partner, residence, birth country), birth characteristics (participant's sex, calendar year of birth).

Model 2: adjusted for medications for maternal autoimmune diseases used before or during pregnancy in addition to variables in Model 1.

Model 3: adjusted for medications for maternal mental diseases used before or during pregnancy in addition to variables in Model 2.

© 2022 He H et al. *JAMA Network Open.*

eTable 15. **Hazard ratios of specific mental disorder according to prenatal exposure to any maternal autoimmune disease, adjusted for medications used by mothers before childbirth in addition to the variables in the main model.**

Exposures and outcomes	No of events/rate per 1000 person years	HR (95% CI), crude	HR (95% CI), Model 1	HR (95% CI), Model 2	HR (95% CI), Model 3
**Any mental disorder**					
exposed	2562/8.97	1.28(1.23-1.33)	1.21(1.17-1.26)	1.18(1.13-1.22)	1.16(1.11-1.20)
unexposed	69899/7.56	1.00 (ref)	1.00 (ref)	1.00 (ref)	1.00 (ref)
**Organic disorders**					
exposed	10/0.03	1.61(0.85-3.04)	1.61(0.85-3.03)	1.54(0.81-2.91)	1.53(0.81-2.89)
unexposed	220/0.02	1.00 (ref)	1.00 (ref)	1.00 (ref)	1.00 (ref)
**Substance use disorders**					
exposed	54/0.89	1.17(0.89-1.53)	1.12(0.86-1.47)	1.09(0.83-1.43)	1.09(0.83-1.42)
unexposed	2116/0.87	1.00 (ref)	1.00 (ref)	1.00 (ref)	1.00 (ref)
**Schizophrenia**					
exposed	37/0.61	1.37(0.99-1.90)	1.30(0.94-1.80)	1.28(0.92-1.78)	1.27(0.92-1.77)
unexposed	1178/0.49	1.00 (ref)	1.00 (ref)	1.00 (ref)	1.00 (ref)
**Mood disorders**					
exposed	85/1.41	1.14(0.92-1.42)	1.07(0.86-1.32)	1.04(0.84-1.29)	1.03(0.83-1.28)
unexposed	3246/1.34	1.00 (ref)	1.00 (ref)	1.00 (ref)	1.00 (ref)
**Neurotic disorders**					
exposed	565/3.43	1.45(1.33-1.57)	1.33(1.22-1.44)	1.27(1.17-1.38)	1.24(1.14-1.35)
unexposed	15555/2.68	1.00 (ref)	1.00 (ref)	1.00 (ref)	1.00 (ref)
**OCD**					
exposed	88/0.53	1.53(1.24-1.90)	1.42(1.15-1.76)	1.37(1.11-1.70)	1.34(1.09-1.67)
unexposed	2195/0.38	1.00 (ref)	1.00 (ref)	1.00 (ref)	1.00 (ref)
**Eating disorders**					
exposed	90/0.3	1.24(1.01-1.53)	1.17(0.95-1.44)	1.13(0.92-1.40)	1.11(0.90-1.37)
unexposed	2777/0.29	1.00 (ref)	1.00 (ref)	1.00 (ref)	1.00 (ref)
**Personality disorders**					
exposed	19/0.31	0.98(0.62-1.54)	0.90(0.57-1.41)	0.85(0.54-1.34)	0.84(0.53-1.33)
unexposed	880/0.36	1.00 (ref)	1.00 (ref)	1.00 (ref)	1.00 (ref)
**Intellectual disability**					
exposed	157/0.53	1.17(1.00-1.37)	1.21(1.03-1.41)	1.18(1.01-1.39)	1.18(1.00-1.38)
unexposed	4347/0.46	1.00 (ref)	1.00 (ref)	1.00 (ref)	1.00 (ref)
**Developmental disorders**					
**exposed**	570/1.94	1.28(1.18-1.39)	1.18(1.09-1.29)	1.16(1.07-1.26)	1.14(1.05-1.24)
unexposed	15110/1.59	1.00 (ref)	1.00 (ref)	1.00 (ref)	1.00 (ref)
**Childhood autism**					
exposed	253/0.86	1.33(1.17-1.51)	1.23(1.08-1.39)	1.21(1.07-1.38)	1.19(1.05-1.36)
unexposed	6166/0.65	1.00 (ref)	1.00 (ref)	1.00 (ref)	1.00 (ref)
**Behavioral disorders**					
exposed	1301/4.47	1.28(1.21-1.36)	1.22(1.16-1.29)	1.18(1.11-1.24)	1.16(1.09-1.22)
unexposed	34698/3.69	1.00 (ref)	1.00 (ref)	1.00 (ref)	1.00 (ref)
**ADHD**					
exposed	735/3.29	1.32(1.23-1.43)	1.25(1.16-1.34)	1.20(1.11-1.29)	1.18(1.10-1.27)
unexposed	19437/2.59	1.00 (ref)	1.00 (ref)	1.00 (ref)	1.00 (ref)
**ODD/CD**					
exposed	106/0.47	1.10(0.90-1.33)	1.10(0.90-1.33)	1.06(0.87-1.29)	1.05(0.86-1.27)
unexposed	3335/0.44	1.00 (ref)	1.00 (ref)	1.00 (ref)	1.00 (ref)

HR=Hazard Ratio, CI=Confidential Interval, OCD=Obsessive-Compulsive Disorder, ADHD=Attention Deficit/Hyperactivity Disorder, ODD/CD=oppositional defiant disorder/conduct disorder.

Model 1: adjusted for parental psychiatric history, maternal characteristics (parity, age at birth, highest education level, cohabitation with a partner, residence, birth country), birth characteristics (participant’s sex, calendar year of birth).

Model 2: adjusted for medications for maternal autoimmune diseases used before or during pregnancy in addition to variables in Model 1.

Model 3: adjusted for medications for maternal mental diseases used before or during pregnancy in addition to variables in Model 2.

© 2022 He H et al. *JAMA Network Open.*

eTable 16. **Associations between specific maternal autoimmune diseases diagnosed before childbirth and overall mental disorders in offspring, with multiple imputation for missing data.**

**Exposures**	**No of events**	**rate per 1000 person years**	**HR (95% CI), crude**	**HR (95% CI), adjusted[a]**
Any autoimmune diseases	5460	9.38	1.41 (1.37-1.45)	1.17 (1.13-1.20)
Endocrine autoimmune diseases	2140	9.51	1.37 (1.31-1.43)	1.19 (1.14-1.24)
Type 1 Diabetes	1494	10.28	1.32 (1.25-1.39)	1.24 (1.18-1.30)
Thyreotoxicosis	545	8.16	1.50 (1.38-1.63)	1.08 (0.99-1.17)
Autoimmune thyroiditis	98	7.79	1.70 (1.40-2.07)	1.16 (0.95-1.41)
Primary adrenocortical insufficiency	35	10.44	1.51 (1.09-2.11)	1.29 (0.92-1.79)
Connective tissue autoimmune diseases	798	10.09	1.60 (1.49-1.71)	1.24 (1.16-1.33)
Rheumatoid arthritis	403	9.75	1.57 (1.42-1.73)	1.25 (1.14-1.38)
Juvenile arthritis	203	10.58	1.73 (1.51-1.99)	1.16 (1.01-1.33)
Dermatopolymyositis	15	7.29	1.07 (0.65-1.78)	0.83 (0.50-1.38)
Polymyalgia rheumatica/temporal arteritis	8	20.08	4.75 (2.38-9.46)	2.85 (1.43-5.71)
Scleroderma	30	11.06	1.79 (1.25-2.55)	1.35 (0.94-1.92)
Systemic lupus erythematosus	125	10.83	1.65 (1.38-1.96)	1.35 (1.13-1.60)
Sjögren syndrome	27	9.82	1.80 (1.24-2.62)	1.26 (0.86-1.84)
Ankylosing Spondylitis	67	8.85	1.43 (1.12-1.81)	1.14 (0.90-1.45)
Wegener granulomatosis	<6	7.39	1.27 (0.48-3.35)	0.97 (0.36-2.58)
Gastrointestinal autoimmune diseases	1520	8.75	1.38 (1.31-1.45)	1.11 (1.05-1.17)
Celiac disease	106	9.38	1.72 (1.42-2.07)	1.16 (0.96-1.40)
Crohn disease	576	8.96	1.43 (1.32-1.55)	1.09 (1.00-1.18)
Ulcerative colitis	894	8.16	1.27 (1.19-1.36)	1.06 (0.99-1.13)
Primary biliary cirrhosis	12	22.15	4.28 (2.45-7.47)	3.09 (1.75-5.44)
Autoimmune hepatitis	76	13.1	2.03 (1.62-2.54)	1.52 (1.21-1.90)
Blood autoimmune diseases	138	9.14	1.47 (1.24-1.73)	1.11 (0.94-1.31)
Pernicious anemia	43	11.25	1.92 (1.42-2.58)	1.32 (0.98-1.79)
Autoimmune hemolytic anemia	10	7.75	1.26 (0.68-2.32)	0.91 (0.49-1.69)
Idiopathic thrombocyopenic purpura	87	8.56	1.34 (1.09-1.65)	1.06 (0.86-1.30)
Nervous system autoimmune diseases	434	9.53	1.41 (1.28-1.55)	1.18 (1.07-1.29)
Multiple sclerosis	293	9.46	1.45 (1.30-1.63)	1.22 (1.08-1.36)
Guillanian-Barré syndrome	116	10.56	1.44 (1.20-1.72)	1.18 (0.98-1.41)
Myasthenia gravis	30	7.42	1.00 (0.69-1.44)	0.89 (0.62-1.27)
Skin autoimmune diseases	578	10.31	1.56 (1.44-1.70)	1.21 (1.11-1.31)
Pemphigus	7	10.6	1.88 (0.91-3.91)	1.20 (0.57-2.52)
Pemphigoid	<6	10.5	1.77 (0.67-4.68)	1.12 (0.42-2.99)
Psoriasis vulgaris	491	11	1.63 (1.49-1.78)	1.26 (1.16-1.38)
Alopecia areata	56	7.85	1.25 (0.96-1.61)	0.99 (0.76-1.29)
Vitiligo	24	6.79	1.23 (0.83-1.82)	0.90 (0.61-1.35)

HR=Hazard Ratio, CI=Confidential Interval

[a]HRs were adjusted for parental psychiatric history, maternal characteristics (parity, age at birth, highest education level, cohabitation with a partner, residence, birth country) and birth characteristics (participant's sex, calendar year of birth)

© 2022 He H et al. *JAMA Network Open.*

eTable 17. **Hazard ratios of specific mental disorder according to prenatal exposure to any maternal autoimmune disease, with multiple imputation for missing data**

Exposures and outcomes	No of events	rate per 1000 person years	HR (95% CI), crude	HR (95% CI), adjusted[a]
**Any mental disorder**				
exposed	5460	9.38	1.41 (1.37-1.45)	1.17 (1.13-1.20)
unexposed	303092	7.91	1.00 (ref)	1.00 (ref)
**Organic disorders**				
exposed	71	0.11	1.56 (1.23-1.97)	1.54 (1.22-1.95)
unexposed	4117	0.10	1.00 (ref)	1.00 (ref)
**Substance use disorders**				
exposed	854	3.24	1.10 (1.03-1.18)	1.05 (0.99-1.13)
unexposed	73465	3.21	1.00 (ref)	1.00 (ref)
**Schizophrenia**				
exposed	319	1.19	1.54 (1.38-1.72)	1.36 (1.22-1.52)
unexposed	20171	0.86	1.00 (ref)	1.00 (ref)
**Mood disorders**				
exposed	657	2.47	1.29 (1.19-1.39)	1.13 (1.04-1.22)
unexposed	51512	2.22	1.00 (ref)	1.00 (ref)
**Neurotic disorders**				
exposed	1723	4.01	1.46 (1.40-1.53)	1.22 (1.16-1.28)
unexposed	107385	3.37	1.00 (ref)	1.00 (ref)
**OCD**				
exposed	213	0.49	1.84 (1.60-2.10)	1.42 (1.24-1.63)
unexposed	9623	0.30	1.00 (ref)	1.00 (ref)
**Eating disorders**				
exposed	257	0.42	1.31 (1.16-1.49)	1.13 (0.99-1.27)
unexposed	16255	0.40	1.00 (ref)	1.00 (ref)
**Personality disorders**				
exposed	374	1.40	1.23 (1.11-1.36)	1.11 (1.00-1.23)
unexposed	30538	1.31	1.00 (ref)	1.00 (ref)
**Intellectual disability**				
exposed	307	0.50	1.27 (1.13-1.42)	1.18 (1.06-1.33)
unexposed	14284	0.35	1.00 (ref)	1.00 (ref)
**Developmental disorders**				
exposed	755	1.23	1.73 (1.61-1.86)	1.13 (1.05-1.22)
unexposed	27272	0.67	1.00 (ref)	1.00 (ref)
**Childhood autism**				
exposed	299	0.48	1.94 (1.73-2.18)	1.20 (1.07-1.35)
unexposed	8689	0.21	1.00 (ref)	1.00 (ref)
**Behavioral disorders**				
exposed	1998	3.30	1.58 (1.51-1.65)	1.19 (1.13-1.24)
unexposed	80984	2.01	1.00 (ref)	1.00 (ref)
**ADHD**				
exposed	1099	2.12	1.74 (1.64-1.85)	1.18 (1.11-1.25)
unexposed	43929	1.21	1.00 (ref)	1.00 (ref)
**ODD/CD**				
exposed	10766	0.29	1.31 (1.15-1.50)	1.12 (0.98-1.28)
unexposed	219	0.42	1.00 (ref)	1.00 (ref)

HR=Hazard Ratio, CI=Confidential Interval, OCD=Obsessive-Compulsive Disorder, ADHD=Attention Deficit/Hyperactivity Disorder, ODD/CD=oppositional defiant disorder/conduct disorder.

[a]HRs were adjusted for parental psychiatric history, maternal characteristics (parity, age at birth, highest education level,

© 2022 He H et al. *JAMA Network Open.*

cohabitation with a partner, residence, birth country) and birth characteristics (participant's sex, calendar year of birth)

eTable 18. **Associations between specific maternal autoimmune diseases diagnosed before childbirth and overall mental disorders in offspring, using propensity score methods.**

Exposures	HR (95% CI), model 1	HR (95% CI), model 2	HR (95% CI), model 3
Any autoimmune diseases	1.16 (1.13-1.19)	1.18 (1.15-1.20)	1.19 (1.16-1.22)
Endocrine autoimmune diseases	1.19 (1.14-1.24)	1.21(1.17-1.26)	1.23(1.18-1.28)
Type 1 Diabetes	1.24 (1.18-1.30)	1.24 (1.17-1.30)	1.25 (1.19-1.32)
Thyreotoxicosis	1.08 (0.99-1.17)	1.03 (0.97-1.09)	1.10 (1.01-1.20)
Autoimmune thyroiditis	1.18 (0.97-1.44)	1.10 (0.97-1.25)	1.26 (1.03-1.54)
Primary adrenocortical insufficiency	1.27 (0.91-1.77)	1.28 (0.98-1.69)	1.30 (0.93-1.81)
Connective tissue autoimmune diseases	1.24 (1.16-1.33)	1.22 (1.16-1.29)	1.27 (1.19-1.36)
Rheumatoid arthritis	1.25 (1.14-1.38)	1.19 (1.10-1.28)	1.27 (1.16-1.41)
Juvenile arthritis	1.15 (1.00-1.32)	1.07 (0.95-1.22)	1.18 (1.03-1.35)
Dermatopolymyositis	0.82 (0.50-1.36)	0.90 (0.57-1.41)	1.01 (0.61-1.67)
Polymyalgia rheumatica/temporal arteritis	2.82 (1.41-5.64)	4.69 (1.92-11.49)	3.50 (1.75-7.00)
Scleroderma	1.33 (0.93-1.90)	1.89 (1.43-2.48)	1.45 (1.02-2.08)
Systemic lupus erythematosus	1.34 (1.13-1.60)	1.44 (1.25-1.65)	1.35 (1.14-1.61)
Sjögren syndrome	1.27 (0.87-1.85)	0.91 (0.64-1.29)	1.33 (0.91-1.94)
Ankylosing Spondylitis	1.15 (0.90-1.46)	1.13 (0.94-1.35)	1.20 (0.94-1.52)
Wegener granulomatosis	0.94 (0.35-2.50)	0.78 (0.22-2.82)	0.93 (0.35-2.48)
Gastrointestinal autoimmune diseases	1.11 (1.05-1.17)	1.06 (1.02-1.10)	1.12 (1.06-1.18)
Celiac disease	1.17 (0.96-1.41)	1.02 (0.89-1.18)	1.21(1.00-1.47)
Crohn disease	1.08 (1.00-1.17)	1.24 (1.17-1.32)	1.10 (1.01-1.19)
Ulcerative colitis	1.07 (1.00-1.14)	0.97 (0.92-1.03)	1.07 (1.00-1.14)
Primary biliary cirrhosis	3.07 (1.75-5.41)	3.83 (1.83-7.99)	3.47 (2.00-6.04)
Autoimmune hepatitis	1.50 (1.20-1.88)	1.63 (1.31-2.03)	1.75 (1.40-2.20)
Blood autoimmune diseases	1.10 (0.93-1.30)	1.18 (1.03-1.34)	1.13 (0.95-1.33)
Pernicious anemia	1.31 (0.98-1.77)	1.45 (1.11-1.89)	1.34 (1.00-1.81)
Autoimmune hemolytic anemia	0.91 (0.49-1.69)	1.60 (1.03-2.50)	0.90 (0.48-1.67)
Idiopathic thrombocyopenic purpura	1.05 (0.85-1.30)	1.15 (0.98-1.35)	1.08 (0.87-1.33)
Nervous system autoimmune diseases	1.17 (1.07-1.29)	1.17 (1.08-1.27)	1.20 (1.09-1.32)
Multiple sclerosis	1.21 (1.08-1.36)	1.16 (1.06-1.28)	1.23 (1.10-1.38)
Guillanian-Barré syndrome	1.17 (0.98-1.41)	1.39 (1.17-1.65)	1.20 (1.00-1.44)
Myasthenia gravis	0.90 (0.63-1.28)	0.94 (0.67-1.31)	0.89 (0.63-1.28)
Skin autoimmune diseases	1.19 (1.10-1.29)	1.23 (1.15-1.32)	1.22 (1.13-1.33)
Pemphigus	1.15 (0.55-2.42)	1.75 (0.78-3.94)	1.28 (0.61-2.69)
Pemphigoid	1.09 (0.41-2.89)	1.87 (0.75-4.65)	1.11 (0.42-2.96)
Psoriasis vulgaris	1.24 (1.14-1.36)	1.21 (1.12-1.31)	1.27 (1.16-1.39)
Alopecia areata	0.99 (0.76-1.29)	1.19 (0.98-1.43)	1.02 (0.79-1.33)
Vitiligo	0.91 (0.61-1.36)	1.14 (0.84-1.54)	0.90 (0.60-1.35)

HR=Hazard Ratio, CI=Confidential Interval

Model 1: adjusted for parental psychiatric history, maternal characteristics (parity, age at birth, highest education level, cohabitation with a partner, residence, birth country) and birth characteristics (participant's sex, calendar year of birth)

Model 2: applied inverse probability weighting approach.

Model 3: adjusted propensity score directly

© 2022 He H et al. *JAMA Network Open.*

eTable 19. **Hazard ratios of specific mental disorder among individuals born to mothers with any autoimmune disease, using propensity score methods.**

Exposures and outcomes	HR (95% CI), model 1	HR (95% CI), model 2	HR (95% CI), model 3
**Any mental disorder**			
exposed	1.16 (1.13-1.19)	1.18 (1.15-1.20)	1.19 (1.16-1.22)
unexposed	1.00 (ref)	1.00 (ref)	1.00 (ref)
**Organic disorders**			
exposed	1.54 (1.21-1.94)	1.55 (1.31-1.82)	1.62 (1.28-2.06)
unexposed	1.00 (ref)	1.00 (ref)	1.00 (ref)
**Substance use disorders**			
exposed	1.05 (0.98-1.12)	1.11 (1.07-1.17)	1.11 (1.04-1.18)
unexposed	1.00 (ref)	1.00 (ref)	1.00 (ref)
**Schizophrenia**			
exposed	1.35 (1.21-1.51)	1.47 (1.36-1.59)	1.41 (1.26-1.57)
unexposed	1.00 (ref)	1.00 (ref)	1.00 (ref)
**Mood disorders**			
exposed	1.12 (1.04-1.21)	1.17 (1.11-1.24)	1.15 (1.07-1.24)
unexposed	1.00 (ref)	1.00 (ref)	1.00 (ref)
**Neurotic disorders**			
exposed	1.21 (1.16-1.27)	1.21 (1.17-1.25)	1.24 (1.18-1.30)
unexposed	1.00 (ref)	1.00 (ref)	1.00 (ref)
**OCD**			
exposed	1.42 (1.24-1.63)	1.42 (1.27-1.58)	1.45 (1.26-1.66)
unexposed	1.00 (ref)	1.00 (ref)	1.00 (ref)
**Eating disorders**			
exposed	1.12 (0.99-1.27)	1.12 (1.02-1.24)	1.13 (1.00-1.28)
unexposed	1.00 (ref)	1.00 (ref)	1.00 (ref)
**Personality disorders**			
exposed	1.10 (1.00-1.22)	1.22 (1.14-1.30)	1.17 (1.06-1.30)
unexposed	1.00 (ref)	1.00 (ref)	1.00 (ref)
**Intellectual disability**			
exposed	1.19 (1.07-1.34)	1.32 (1.20-1.45)	1.25 (1.12-1.40)
unexposed	1.00 (ref)	1.00 (ref)	1.00 (ref)
**Developmental disorders**			
exposed	1.14 (1.06-1.23)	1.14 (1.06-1.22)	1.17 (1.09-1.26)
unexposed	1.00 (ref)	1.00 (ref)	1.00 (ref)
**Childhood autism**			
exposed	1.21(1.08-1.36)	1.23 (1.08-1.39)	1.23 (1.10-1.38)
unexposed	1.00 (ref)	1.00 (ref)	1.00 (ref)
**Behavioral disorders**			
exposed	1.19 (1.14-1.25)	1.23 (1.18-1.28)	1.22 (1.17-1.28)
unexposed	1.00 (ref)	1.00 (ref)	1.00 (ref)
**ADHD**			
exposed	1.19 (1.12-1.26)	1.20 (1.13-1.27)	1.23 (1.16-1.30)
unexposed	1.00 (ref)	1.00 (ref)	1.00 (ref)
**ODD/CD**			
exposed	1.13 (0.98-1.29)	1.28 (1.14-1.43)	1.18 (1.03-1.35)
unexposed	1.00 (ref)	1.00 (ref)	1.00 (ref)

HR=Hazard Ratio, CI=Confidential Interval, OCD=Obsessive-Compulsive Disorder, ADHD=Attention Deficit/Hyperactivity Disorder, ODD/CD=oppositional defiant disorder/conduct disorder.

Model 1: adjusted for parental psychiatric history, maternal characteristics (parity, age at birth, highest education level, cohabitation with a partner, residence, birth country) and birth characteristics (participant's sex, calendar year of birth)

Model 2: applied inverse probability weighting approach.

Model 3: adjusted propensity score directly

© 2022 He H et al. *JAMA Network Open.*

eTable 20. **Association between overall maternal autoimmune diseases diagnosed before childbirth and suicide in offspring.**

**exposures and outcomes**	**No of events**	**rate per 1000 person years**	**HR (95% CI), crude**	**HR (95% CI), adjusted[a]**
**Suicide**				
Exposed	33	0.05	1.35 (0.96-1.90)	1.11 (0.79-1.57)
unexposed	2317	0.06	1.00 (ref)	1.00 (ref)

HR=Hazard Ratio, CI=Confidential Interval.

[a]HRs were adjusted for parental psychiatric history, maternal characteristics (parity, age at birth, highest education level, cohabitation with a partner, residence, birth country) and birth characteristics (participant's sex, calendar year of birth).

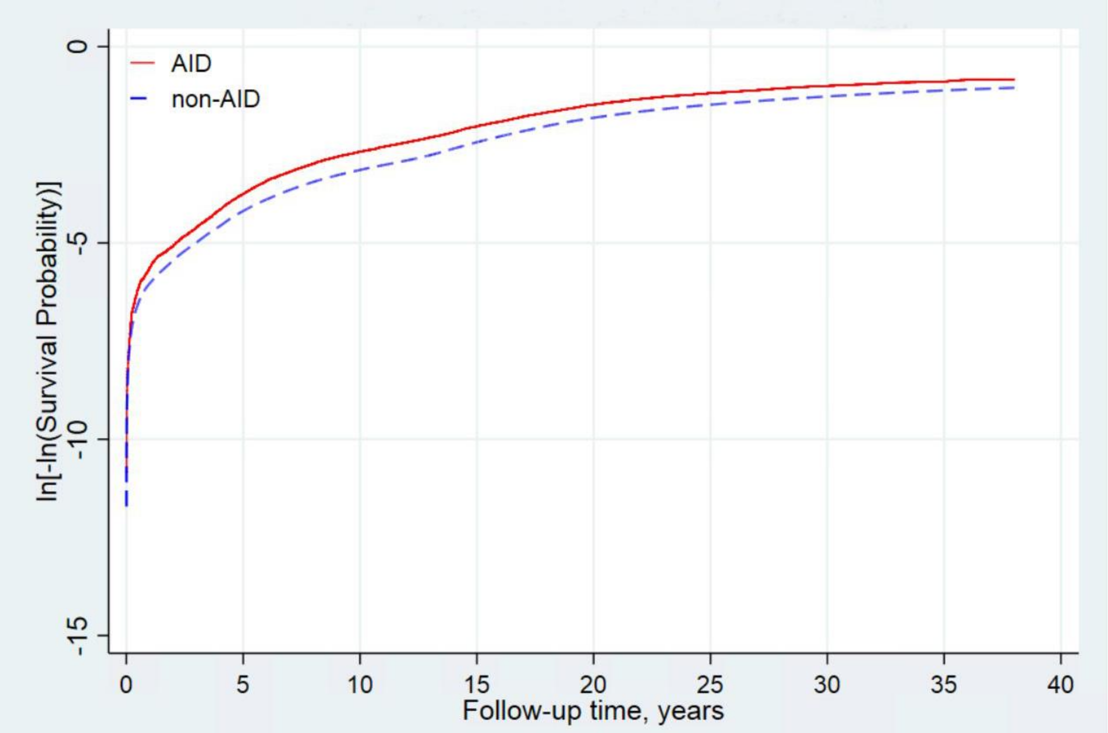

eFigure 1. **The log-minus-log survival curve. AID=autoimmune disease**

© 2022 He H et al. *JAMA Network Open*.
